# Directional Entropy Bands for Surface Characterization of Polymer Crystallization

**DOI:** 10.3390/polym17172399

**Published:** 2025-09-03

**Authors:** Elyar Tourani, Brian J. Edwards, Bamin Khomami

**Affiliations:** Materials Research and Innovation Laboratory, Department of Chemical and Biomolecular Engineering, University of Tennessee, Knoxville, TN 37996, USA; etourani@vols.utk.edu

**Keywords:** polymer crystallization, directional entropy bands, local order parameters, nucleation, surface detection, molecular dynamics, phase transitions

## Abstract

Molecular dynamics (MD) simulations provide atomistic insights into nucleation and crystallization in polymers, yet interpreting their complex spatiotemporal data remains a challenge. Existing order parameters face limitations, such as failing to account for directional alignment or lacking sufficient spatial resolution, preventing them from accurately capturing the anisotropic and heterogeneous characteristics of nucleation or the surface phenomena of polymer crystallization. We introduce a novel set of local order parameters—namely, *directional entropy bands*— that extend scalar entropy-based descriptors by capturing first-order angular moments of the local entropy field around each particle. We compare these against conventional metrics (entropy, the crystallinity index, and smooth overlap of atomic positions (SOAP) descriptors) in equilibrium MD simulations of polymer crystallization. We show that (i) scalar entropy bands demonstrate advantages compared to SOAP in polymer phase separation at single-snapshot resolution and (ii) directional extensions (dipole projections and gradient estimates) robustly highlight the evolving crystal–melt interface, enabling earlier nucleation detection and quantitative surface profiling. UMAP embeddings of these 24–30D feature vectors reveal a continuous melt–surface–core manifold, as confirmed by supervised boundary classification. Our approach is efficient and directly interpretable, offering a practical framework for studying polymer crystallization kinetics and surface growth phenomena.

## 1. Introduction

Crystallization in polymers is fundamentally more complex than in small molecules due to chain connectivity and topological constraints, which necessitate both configurational and spatial ordering. Upon cooling, polymers transition from coiled to semicrystalline states, yet a persistent amorphous phase often remains [1,2,3,4,5]. This transformation is driven by weak van der Waals interactions, whereas excluded volume effects hinder entropic collapse into crystalline configurations [6]. Classical models such as the Lauritzen–Hoffman (LH) [7] and Sadler–Gilmer (SD) [8] models have described lamellar growth, but several aspects remain unresolved: the role of the local structure in the melt prior to nucleation, the dynamic re-entry of chains into crystalline regions, and how heterogeneous liquid environments influence growth pathways [9,10,11].

Molecular simulations have helped clarify nonequilibrium crystallization mechanisms in short [12,13,14] and entangled chains [15,16,17,18,19,20], often using local order parameters. These descriptors are designed to extract physically meaningful structures from disordered polymer configurations using rotation- and translation-invariant representations [21,22]. Angle-based alignment metrics, commonly applied to quantify segmental order, classify crystalline domains by comparing the principal axes of adjacent segments [12,14,23,24,25]. However, angle-based metrics lack universal thresholds and are undefined at chain ends, and spatial averaging, though useful for noise reduction, often obscures interfacial and precursor structures critical to nucleation and growth.

A range of translational and orientational order parameters (OPs) have been developed for identifying structural motifs in small-molecule systems, including Common Neighbor Analysis (CNA) [26], Bond Angle Analysis (BAA) [27], the Centrosymmetry Parameter (CNP) [28], Voronoi tessellation [29,30], and Steinhardt’s Bond-Orientational Order (BOO) parameters [31]. Although many of these descriptors were originally designed to detect crystalline phases such as fcc, bcc, and icosahedral structures in atomic systems [21], select methods, particularly the Voronoi and BOO methods, have shown promise for application to polymeric systems. Recently [32], we systematically assessed these OPs in the context of polymer crystallization. Voronoi analysis adapts well to local density variations but lacks information on orientational symmetry. In contrast, BOO parameters capture rotationally invariant local motifs using spherical harmonics [33] and have been used to study crystallization, glass transitions, and interfacial behavior in soft-matter systems [22,34,35,36]. However, conventional BOO calculations are highly sensitive to how neighbors are defined—by distance, number, or topology—and can fail to resolve subtle structural distinctions, such as variations in Wyckoff positions [37]. Methods such as solid-angle nearest neighbors (SANN) [38] and polyhedral template matching (PTM) [39] have addressed some of these limitations in small-molecule systems, but their effectiveness in polymers remains limited. In particular, the search for unique order parameters to identify and classify potentially short-lived precursor states has not yet been successful.

Spectrum-based descriptors, such as Smooth Overlap of Atomic Positions (SOAP) [40], Behler–Parrinello symmetry functions, and bispectrum coefficients, have extended the capabilities of traditional order parameters by mapping local environments into high-dimensional feature spaces. Unlike conventional BOO parameters, which summarize local symmetry using spherical harmonics, these descriptors retain richer structural information and enable advanced machine learning workflows [41,42,43]. To the best of our knowledge, this study presents the first systematic application and evaluation of SOAP descriptors in polymer crystallization, as their ability to capture chain connectivity, local packing anisotropy, and precursor states has not been previously explored.

Machine learning techniques have been increasingly used to extract structure–property relationships from high-dimensional data in a wide range of material applications [22,44,45,46,47]. Spectrum-based descriptors have been widely used in atomic systems to investigate crystallization, phase behavior, and self-assembly through unsupervised learning and clustering techniques, often combined with dimensionality reduction [48,49,50]. In particular, Spellings and Glotzer [41] introduced a fingerprint that avoids spherical harmonic averaging, using odd *l* values and variable neighborhood sizes to enhance rotational sensitivity. Adorf et al. [42] further integrated bispectrum features with BOO, bond-angle, and distance metrics for manifold learning via HDBSCAN and classifier training. More recently, Bhardwaj et al. [51] applied self-supervised autoencoders to analyze conformational fingerprints constructed from shell-averaged angle-based OPs, enabling the detection of local order in entangled polymer systems.

Parallel to geometric and symmetry-based descriptors, local thermodynamic-like parameters—specifically, entropy and enthalpy—have shown strong potential for identifying phase transitions at the particle level. Building on the early statistical mechanics work by Nettleton and Green [52], Piaggi et al. [53,54] introduced localized entropy and enthalpy measures derived from smoothed radial distribution functions, successfully distinguishing liquid and crystalline regions in atomic systems. Unlike ensemble-averaged thermodynamic quantities, these local estimates capture spatial variations relevant to nucleation. Nafar Sefiddashti et al. [19,55] extended this framework to polymer crystallization under elongational flow, demonstrating that configurational entropy could signal the onset of crystallization without requiring system-specific hyperparameters. However, challenges remain in quiescent systems, where radial distribution-based entropy may misclassify folded regions due to their proximity but lack of geometric symmetry. Although folds often appear entropically ordered, they lack orientational coherence, and scalar entropy values cannot fully resolve this distinction.

Recognizing these limitations, in our previous study [32], we developed a machine learning framework that quantitatively assessed a broad set of geometric, entropic, and symmetry-based order parameters in polymer crystallization. Our results showed that scalar entropy provided relatively strong sensitivity to early nucleation events, but its effectiveness diminished in the resolution of the surface structure or the distinction between competing internal motifs. To address this, we introduced the crystallinity index (C-index) [32] to evaluate OP sensitivity in the nucleation stages and identified entropy as one of the most informative indicators of the early stages. These insights motivate a new direction, moving beyond scalar thermodynamic variables toward spatially resolved entropy-based features that retain physical interpretation while capturing configurational anisotropy.

To overcome the limitations of scalar entropy and enhance local structural resolution, we introduce a new class of thermodynamic-like descriptors—namely, directional entropy bands (DEB). This approach extends the idea of local entropy by partitioning the radial space around each atom into concentric shells, or “bands”, and computing the entropy contributions within each band separately. These directional bands capture anisotropic variations in local configurational order and enable a more detailed distinction between folded segments, crystal surfaces, amorphous regions, and precursor structures. By combining radial decomposition with angular moments and dimensionality reduction, this method produces spatially resolved fingerprints that are both interpretable and physically grounded. Importantly, we demonstrate that simple averages of directional entropy bands, when combined with local enthalpy or spatial orientation metrics, can match or exceed the discriminative power of high-dimensional spectral descriptors like SOAP, particularly in early-stage interfacial detection in polymer crystallization. At the same time, SOAP remains advantageous for capturing fine intrachain conformational distinctions such as gauche–trans separation.

The interfacial features highlighted by DEB are consistent with the three-phase morphology of semicrystalline, crystal, mobile amorphous, and rigid amorphous polymers, as established by calorimetry and solid-state NMR [56,57]. Atomic force microscopy (AFM) and scattering (SAXS/WAXS) further reveal lamellar stacking, amorphous layer thickness, and the specific crystal–amorphous interfacial area during growth [58], which is analogous to the interfacial sensitivity captured by our surface-weighted entropy gradient. Although demonstrated here for polyethylene, DEB is a general framework and could, in principle, be extended to other semicrystalline polymers, including conjugated systems where π–π stacking and orientational order are central. Recent studies on conjugated polymers highlight the importance of such structural features–for example, probing how dopants influence molecular stacking in crystalline polymers [59] or elucidating atomic-scale mechanisms of semicrystalline orientation and π–π stacking distances in detail [60], which DEB could complement in future simulation-based analyses. While this paper remains methodological, these connections suggest that DEB provides a foundation for linking atomistic simulations to experimental observations across a broad class of semicrystalline polymers.

This work makes several key contributions: (i) It introduces directional entropy bands as a novel family of thermodynamic-like order parameters tailored for polymer crystallization. (ii) It benchmarks the performance of these descriptors against conventional OPs and spectrum-based ML fingerprints across nucleation regimes. (iii) It proposes a simple yet effective averaging scheme that combines entropy bands with angular information to enhance the identification of anisotropic structures, such as folds and crystal edges. (iv) It provides a machine learning workflow that integrates unsupervised and supervised methods to validate the effectiveness of entropy bands in phase classification.

The remainder of the paper is organized as follows. Section 2 presents the theoretical foundation and computational methodology, including the calculation of entropy bands, angular expansions, and ML workflows. Section 3 evaluates the performance of entropy bands through dimensionality reduction, clustering, and classification, comparing them with conventional and spectrum-based OPs, and discusses the implications of directional entropy in identifying precursor structures and interfacial features. Finally, Section 4 summarizes the key findings and provides directions for future work.

## 2. Materials and Methods

### 2.1. Molecular Dynamics Simulation Details

Molecular dynamics simulations were performed using the LAMMPS package [61,62], employing the Siepmann–Karaboni–Smit (SKS) unified atom (UA) model [63] to represent polyethylene chains. In this model, each CH_2_ group is treated as a single interaction site, while the CH_3_ terminal groups serve as head and tail beads. To improve numerical stability and eliminate the need for rigid bond constraints in integration, the original constraint-based bonds were replaced with harmonic potentials [64,65,66,67,68]. Although some uncertainty remains about the ability of UA force fields to reproduce true crystal microstructures in the ground state of polyethylene [69], the SKS model remains well suited to study the dynamics of nucleation and crystallization [19,70].

Non-bonded interactions were modeled using a standard 12–6 Lennard–Jones (LJ) potential [63]:

(1) $$U_{\mathrm{LJ}}\left(r_{ij}\right)=4\epsilon_{ij}\left[{\left(\frac{\sigma_{ij}}{r_{ij}}\right)}^{12}-{\left(\frac{\sigma_{ij}}{r_{ij}}\right)}^{6}\right],$$

where $\epsilon_{ij}$ is the potential well depth and $\sigma_{ij}$ is the zero-crossing distance. The parameter values were set as $\epsilon_{i}/k_{B}=47\;K$ and $\sigma_{i}=3.93\;Å$ for the CH_2_ and $\epsilon_{i}/k_{B}=114\;K$ for the CH_3_ groups. Interactions between different atom types were calculated using the Lorentz–Berthelot mixing rules: $\epsilon_{ij}=\sqrt{\epsilon_{i}\epsilon_{j}}$ and $\sigma_{ij}=\left(\sigma_{i}+\sigma_{j}\right)/2$. Lennard–Jones interactions within chains were excluded for atoms separated by fewer than four bonds, and all LJ interactions were truncated at a cutoff of $2.5\;\sigma_{i}$.

Bonded interactions including harmonic bond stretching were quantified by

(2) $$U_{\mathrm{str}}\left(l\right)=\frac{k_{l}}{2}{\left(l-l_{0}\right)}^{2},$$

with an equilibrium bond length of $l_{0}=1.54\;Å$ and force constant of $k_{l}/k_{B}=452,900\;{K/Å}^{2}$. Harmonic angle bending was described by

(3) $$U_{\mathrm{bend}}\left(\theta \right)=\frac{k_{\theta }}{2}{\left(\theta -\theta_{0}\right)}^{2},$$

with an equilibrium angle of $\theta_{0}=114^{\circ }$ and $k_{\theta }/k_{B}=62,500\;{K/\mathrm{rad}}^{2}$. Torsional interactions were defined by

(4) $$U_{\mathrm{tor}}\left(\phi \right)=\sum_{m=0}^{3}a_{m}\cos^{m}\phi ,$$

with coefficients of $a_{0}/k_{B}=1010$, $a_{1}/k_{B}=-2019$, $a_{2}/k_{B}=136.4$, and $a_{3}/k_{B}=3165\;K$ [19,63,71].

Simulations were performed under isothermal and isobaric (NPT) conditions at 1 atm with orthorhombic periodic boundaries. Temperature and pressure were controlled using a Nosé–Hoover thermostat and barostat. The system consisted of 60 polyethylene chains of n-pentacontahectane (C150), modeled at a quench temperature of 300K, which corresponds to approximately 25% undercooling. This setup is consistent with previous nucleation studies for long-chain polymers [14,17]. Please note that all analyzed descriptors (scalar entropy, directional entropy bands, or SOAP) are functions of instantaneous atomic coordinates. We employed Nosé–Hoover coupling in NPT to stabilize thermodynamic conditions during crystallization, a common choice in polymer MD, since density and volume evolve as the system crystallizes. However, because classification hinges on local structure, we do not expect the structural labels to be sensitive to ensemble choice.

Before quenching, the system was equilibrated at 550K for 200ns to ensure thermal homogeneity, then cooled to 300K, when a single nucleation event was observed. The density evolved from $0.76\;{g/\mathrm{cm}}^{3}$ at 450K to $0.94\;{g/\mathrm{cm}}^{3}$ at the end of crystallization. Details of the equilibrium protocol, quenching process (used to identify the crystallization range and estimate $T_{m}$ by heating and $T_{c}$ by subsequent cooling process) are provided in the Supplementary Material, Section S1.

This work is methodological in nature, focusing on descriptor development and benchmarking. To control system-level variables and highlight the discriminative power of entropy-based features, we selected a specific polymer chemistry and chain length (polyethylene, SKS force field; C150). Although $T_{g}$, the molecular weight, and other parameters are well known to affect semicrystallinity, systematically varying them lies outside our present scope and is reserved for future work. For further validation, we additionally analyze a higher-molecular-weight snapshot (C500) in Supplementary Figure S7.

### 2.2. Order Parameters

#### 2.2.1. Smooth Overlap of Atomic Positions (SOAP) Descriptor

The SOAP descriptor is a density-based atomic environmental characterization that has demonstrated considerable effectiveness in predicting crystalline properties [40,43] and classifying structures [72,73,74]. In a single-species system, specifically a united-atom potential energy model where each site represents an effective methylene group, the single-species SOAP method can be applied. This method [40] describes the local atomic environment using a continuous density function, which is defined as

(5) $$\rho \left(\vec{r}\right)=\sum_{i}\exp\left(-\frac{1}{2\sigma^{2}}{\left|\vec{r}-{\vec{R}}_{i}\right|}^{2}\right)\;,$$

where ${\vec{R}}_{i}$ represents the positions of the atoms and σ is the Gaussian width. The summation encompasses all atoms (*i*) within the local region of interest. To characterize the environment surrounding a selected center (often taken as $\vec{r}=0$ in the atom of interest), one can expand $\rho (\vec{r})$ using a complete basis of radial functions and spherical harmonics as [40]

(6) $$\rho \left(\vec{r}\right)=\sum_{n=1}^{n_{\max}}\sum_{l=0}^{l_{\max}}\sum_{m=-l}^{l}c_{nlm}g_{n}\left(r\right)Y_{lm}\left(\theta ,\phi \right)\;,$$

where $g_{n}\left(r\right)$ represents a set of selected radial basis functions (such as orthonormal radial basis functions, Gaussian-type orbitals), $Y_{lm}\left(\theta ,\phi \right)$ denotes the spherical harmonics, and $c_{nlm}$ represents the expansion coefficients that encapsulate the geometric structure of the local atomic environment. The expansion coefficients ($c_{nlm}$) are calculated [40] as

(7) $$c_{nlm}=\int_{V}\rho \left(\vec{r}\right)g_{n}\left(r\right)Y_{lm}^{*}\left(\theta ,\phi \right)\;dV\;.$$

This choice of basis is a matter of convention and is computationally more efficient but does not alter the final descriptor. The SOAP descriptor is derived by creating rotationally invariant combinations of the coefficients, commonly known as the power spectrum [40]:

(8) $$p_{nn^{\prime }l}=\sum_{m=-l}^{l}c_{nlm}^{*}c_{n^{\prime }lm}\;.$$

The values of $p_{nn^{\prime }l}$ then serve as the components of the SOAP descriptor for the local environment around the selected atom. This captures the essential geometry of the local atomic environment in a manner that is independent of the chosen coordinate frame. Consequently, the descriptor for each particle has dimensions of $n_{\max}\times n_{\max}^{\prime }\times l_{\max}$.

To compute the SOAP vectors, we focused on the united-atom sites of the polymer chains. Based on the RDF plot of the orthorhombic reference crystal structure, a cutoff radius of 6.0 Å was selected (slightly beyond the third peak) for inclusion in the environmental space of the neighbor list of each site. The SOAP parameters used in this study were chosen as $n_{max}=8$, $l_{max}=8$ to balance resolution and computational cost. The width of the Gaussian smearing functions, which controls the resolution of atomic position encoding, was set to 0.1 Å. All other parameters were kept at their default values, as implemented in the DScribe library. [75].

#### 2.2.2. Thermodynamic-like Parameters for Phase Transitions

Local thermodynamic descriptors—specifically, local enthalpy and configurational entropy—have proven effective in distinguishing between crystalline and amorphous environments on the atomic scale [52,53,54]. The local enthalpy for atom *i* is defined by distributing the potential energy and pressure volume contribution of the system into the atomic components as

(9) $$H_{i}=U_{i}+\frac{pV}{N},$$

where $U_{i}$ is the potential energy of the atom *i*, *p* is the pressure, *V* is the volume of the system, and *N* is the total number of atoms. Then, neighbor-averaged local enthalpy is computed as [19,53]

(10) $${\bar{H}}_{i}=\frac{\sum_{j}H_{j}+H_{i}}{N_{\mathrm{neigh}}+1}\;.$$

The local entropy estimate is based on the two-body excess entropy term ($S_{2}$) from Kirkwood’s expansion of the configurational entropy, which captures up to 90% of the total entropy in dense fluids [53,76,77]:

(11) $$S_{2}=-2\pi \rho k_{B}\int_{0}^{\infty }\left[g(r)\ln g(r)-g(r)+1\right]r^{2}\;dr,$$

where ρ is the number density and g(r) is the radial distribution function. For each atom (*i*), the local entropy ($S_{i}$) is calculated using a smoothed atom-specific radial distribution function ($g^{i}\left(r\right)$) truncated at a finite cutoff ($r_{m}$) [19,53]:

(12) $$S_{i}=-2\pi \rho_{N}k_{B}\int_{0}^{r_{m}}\left[g^{i}\left(r\right)\ln g^{i}\left(r\right)-g^{i}\left(r\right)+1\right]r^{2}\;dr,$$

where

(13) $$g^{i}\left(r\right)=\frac{1}{4\pi \rho_{N}r^{2}}\sum_{j}\frac{1}{\sqrt{2\pi \sigma^{2}}}\exp\left(-\frac{{\left(r-r_{ij}\right)}^{2}}{2\sigma^{2}}\right),$$

and $r_{ij}$ is the distance between the atom (*i*) and the neighbor (*j*), with σ representing the smoothing parameter. The neighborhood-averaged local entropy is expressed as

(14) $${\bar{S}}_{i}=\frac{\sum_{j}S_{j}f\left(r_{ij}\right)+S_{i}}{\sum_{j}f\left(r_{ij}\right)+1},$$

where $f(r_{ij})$ is a switching function that decays from 1 to 0 as $r_{ij}\to r_{c}$.

This scalar entropy framework has been widely applied to characterize polymer crystallization under flow conditions [19,78,79,80,81,82,83]. However, coarse scalar averages provide only isotropic and low-resolution information and are often insufficient in quiescent systems, where radial distribution-based entropy may misclassify folded regions. These regions can appear entropically ordered due to local packing proximity, yet they lack geometric symmetry or orientational coherence.

**Figure 1:**
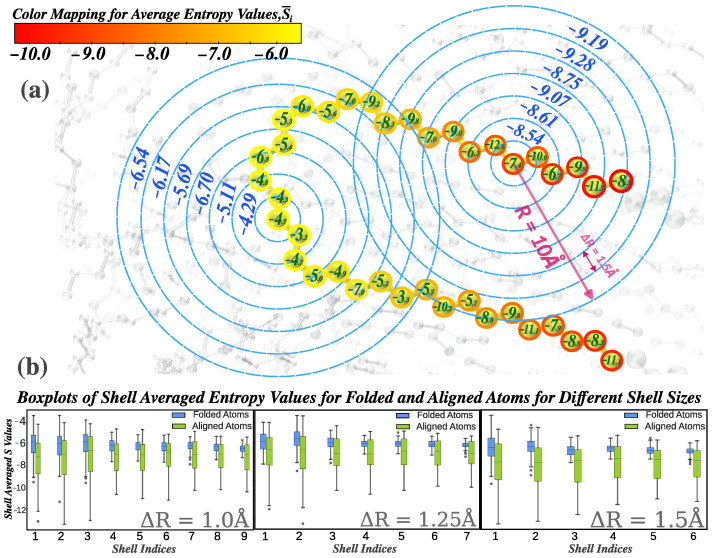
Definition of entropy bands: (**a**) entropy fingerprint vectors for random particles, with the color bar indicating scalar average entropy. Particle labels show non-averaged entropy values, and figure shell labels represent entropy from distinct radial shells (ΔR=1.5); (**b**) box plots of shell-averaged entropy values for randomly selected folded and aligned atoms across varying shell thicknesses (ΔR), highlighting the resolution impact of ΔR.

To overcome this limitation, we introduce an array descriptor termed *entropy bands*, which computes the local entropy within concentric radial shells (Figure 1a). For each atom, the spherical environment is divided into fixed-thickness radial bands (ΔR), and the entropy within each shell is independently averaged, resulting in an entropy fingerprint vector (${\vec{S}}_{i}$). Shell boundaries were selected on the basis of the RDF of a reference orthorhombic crystal structure, with a maximum cutoff of $R_{\max}=10.0\;Å$, previously validated as optimal for phase separation [19]. The fingerprint vector typically includes 6–8 bands, depending on the resolution ΔR, which we varied between 1.0 and 1.5 Å. The value of ΔR=1.5Å was selected for the final analysis due to its slightly superior ability to distinguish folded and aligned structures, as illustrated in Figure 1b.

#### 2.2.3. Directional Entropy Bands

Although entropy bands provide improved spatial resolution compared to scalar entropy, they still represent an isotropic average within each radial shell and are, therefore, limited in their ability to capture orientational features. This isotropy hinders their effectiveness in distinguishing anisotropic structures, such as chain folds, crystallite edges, and precursor motifs, which often exhibit direction-dependent ordering. To overcome this limitation, we introduce *directional entropy bands*, an extension of the entropy-band framework that incorporates angular information through geometric projections of local entropy values.

Rather than explicitly computing a directional radial distribution function (g(r,θ,ϕ)), which is sparse and noisy at the atomic scale, we employ a more tractable and physically interpretable approach. For each atom *i* and the radial band ($\left[r_{1},r_{2}\right]$), the following quantities are calculated based on scalar entropy values of neighboring atoms.

- The *radial entropy average:*
  $${\bar{S}}_{i,r_{1}\to r_{2}}=\frac{1}{N_{\mathrm{shell}}}\sum_{j\in \mathrm{shell}}S_{j},$$
  where $S_{j}$ is the value of the scalar entropy of the neighboring atoms within the shell.
- The *directional entropy projections* along the Cartesian axes:
  $$\langle S_{j}\cdot {\hat{r}}_{ij,x}\rangle ,\;\langle S_{j}\cdot {\hat{r}}_{ij,y}\rangle ,\;\langle S_{j}\cdot {\hat{r}}_{ij,z}\rangle ,$$
  where ${\hat{r}}_{ij}$ is the unit displacement vector from the atom (*i*) to the neighbor (*j*). These dipole-like projections reflect the first-order directional anisotropy of the local entropy field and are analogous to l=1 spherical harmonic components.
- The *max-based entropy gradient* within the shell:
  $$dS_{r_{1}\to r_{2}}^{\max}=\max_{j}\left(\frac{S_{j}-S_{i}}{∥{\vec{r}}_{j}-{\vec{r}}_{i}∥}\right),$$
  which estimates the steepest local change in entropy and is particularly sensitive to interfacial regions and phase boundaries.

The full directional entropy fingerprint for each atom is constructed by concatenating the outputs from all radial shells, including: (i) scalar entropy averages, (ii) directional projections, and (iii) max-based gradients.

To further enhance the ability to identify surface atoms, i.e., those located at sharp boundaries between crystalline and amorphous regions, we define a feature of the *surface weighted entropy gradient*:

$$f_{i}=\exp\left(-\frac{{\left({\bar{S}}_{i}-S^{*}\right)}^{2}}{2\sigma_{s}^{2}}\right)\cdot \max_{r_{1}\to r_{2}}dS_{r_{1}\to r_{2}}^{\max}\;,$$

where ${\bar{S}}_{i}$ is the mean scalar entropy of the atom (*i*, imported or calculated), $S^{*}$ is a characteristic surface entropy (typically around −5.8 in dimensionless LJ units), and $\sigma_{s}$ defines the width of the entropy window. This feature acts as a smooth, localized filter: it suppresses gradient contributions from atoms deep within crystalline or amorphous regions while enhancing those near interfacial zones. The scalar $f_{i}$ has proven effective in isolating surface atoms, even in systems with fluctuating entropy distributions, and is used later in dimensionality reduction and classification analyses.

All calculations were performed using atomic coordinates and entropy values extracted from molecular dynamics snapshots at specific time steps. Neighbor identification was handled using a periodic cKDTree implementation with a minimum image convention. The radial shells ranged from 0.38 to 2.67 Å, divided into six bands based on the radial distribution function of the orthorhombic crystal reference. A Gaussian broadening parameter of σ=0.1 Å was used in the estimation of entropy, consistent with the prior thermodynamic-like descriptors [19,54].

The resulting directional entropy bands, along with the surface-weighted gradient ($f_{i}$), enable fine-grained resolution of structural anisotropy in polymeric systems. As demonstrated later, these descriptors provide a meaningful separation of crystalline, amorphous, and interfacial atoms in both unsupervised and supervised frameworks.

#### 2.2.4. Machine Learning Workflow

A structured machine learning (ML) workflow was applied to evaluate the performance of directional entropy bands (DEB) and related order parameters in classifying crystalline, amorphous, and interfacial (surface) regions. The pipeline combined both unsupervised and supervised methods.

High-dimensional DEB and SOAP descriptors were separately projected onto low-dimensional manifolds using UMAP [84]. Principal Component Analysis (PCA) [85] was applied beforehand only for SOAP features. UMAP was chosen for its efficiency and ability to preserve local topological structure, making it well suited for identifying structural phase organization. Manhattan distance was used, with 10 neighbors and a minimum distance of zero; detailed tuning procedures are reported in our previous study [32] and in the Supplementary Materials, Section S2.1. HDBSCAN [86] was then applied to detect clusters corresponding to melt, crystal, and interfacial regions in the reduced space. Minimum cluster size and sample thresholds were varied between 0.5–5% and 0.5–2% of the system, respectively, consistent with a validated parameter selection framework [32] and presently in the Supplementary Material, Section S2.2. Sensitivity analyses (see Supplementary Material, Figure S3) show that clustering results are robust across this range, as the dominant separation is between the melt and crystalline states. Surface labels for supervised learning were then assigned by combining the crystallinity index (C-index) [32] to identify crystalline atoms with an α-shape procedure to extract surface atoms.

**Figure 2:**
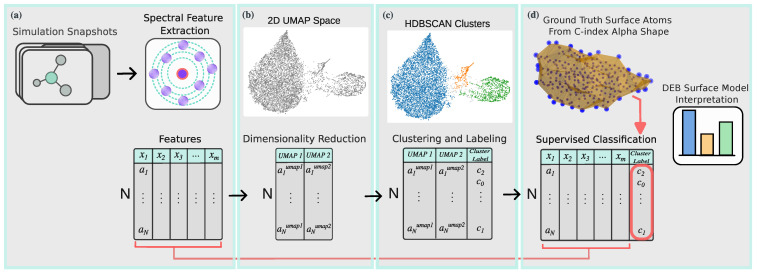
Overview of the ML workflow used to validate directional entropy bands. (**a**) Simulation snapshots are converted to structural features: scalar entropy, entropy bands, DEB, and SOAP (with PCA applied only to SOAP). Note that DEB and SOAP features are analyzed independently. (**b**) Dimensionality reduction with UMAP reveals the low-dimensional manifold of melt, surface, and crystal environments. (**c**) Unsupervised clustering with HDBSCAN assigns data-driven phase labels and highlights transition regions. (**d**) Supervised validation (LogReg, Random Forest, and XGBoost; stratified cross-validation; ROC–AUC) quantifies the predictive power of DEB features.

The unsupervised stage was used to determine latent organization of melt, crystal, and surface states directly from entropy-based descriptors, without bias from predefined labels. In contrast, supervised learning was employed to validate how well DEB features can actually predict structural categories once labeled ground-truth criteria are provided. These supervised models are not introduced as new algorithmic developments; rather, they provide a rigorous test of the discriminative power and interpretability of DEB. For clarity, the complete ML pipeline is summarized in the flowchart of Figure 2. Further implementation details, parameter ranges and evaluation criteria are provided in Section 3. The codes and datasets used for descriptor calculation and machine learning validation (SOAP vectors, DEB feature arrays, scalar entropy values, and associated scripts) are included in the Supplementary Material (SM_DEB_SOAP.zip).

For supervised analysis, Logistic Regression (LogReg), Random Forest, and XGBoost classifiers were trained on structural labels (crystal, melt, and surface) defined based on thermodynamic and geometric criteria. As a linear model, LogReg was applied separately for binary tasks (surface vs. melt, surface vs. crystal), while nonlinear models (RF and XGBoost) were trained for surface vs. non-surface atoms, i.e., melt and crystal atoms within the cluster. Model performance was assessed using stratified cross-validation and evaluated with metrics such as ROC–AUC. Forward Feature Selection (FFS) was employed to identify the most informative DEB features for each classification task. Feature contributions were further interpreted using SHapley Additive exPlanations (SHAP).

## 3. Results and Discussion

To assess the effectiveness of structural order parameters in distinguishing amorphous, crystalline, and interfacial phases during polymer crystallization, we analyze both high-dimensional symmetry-based descriptors and physically interpretable thermodynamic metrics. We begin with the Smooth Overlap of Atomic Positions (SOAP), a geometric fingerprint that encodes local environments via rotationally invariant density projections. As a high-dimensional symmetry-aware descriptor, SOAP has demonstrated strong performance in capturing subtle structural motifs in both crystalline and disordered materials [22,40,43]. Given its sensitivity to local geometric arrangements, we hypothesize that SOAP may also capture interfacial complexity in polymers, particularly in regions where traditional order parameters fail to resolve transitional environments. In the following, after discussing the results of the SOAP method, we will evaluate entropy-based descriptors, beginning with scalar entropy bands; then, we add the directional variants, which integrate the local anisotropy.

Throughout this study, we focus more on four representative simulation time instants to be consistent with the literature [32,51]: pre-nucleation ($t_{\mathrm{pre}}$), transitional ($t_{\mathrm{tr}}$), intermediate growth ($t_{\mathrm{mid}}$), and steady-state crystallization ($t_{\mathrm{ss}}$). These time points were defined in our previous work [32] based on the evolution of system density and cluster size.

### 3.1. Smooth Overlap of Atomic Positions (SOAP) Descriptors

To the best of our knowledge, the use of SOAP in polymeric systems and the findings of the ML analyses to be presented are novel contributions. Therefore, this work is a test application of SOAP descriptors to distinguish structural phases in a polymeric system. In the present case, each SOAP fingerprint vector has 324 components, generated using parameters of $r_{cut}=6.0$, $n_{max}=8$, $l_{max}=8$, and σ=0.1.

**Figure 3:**
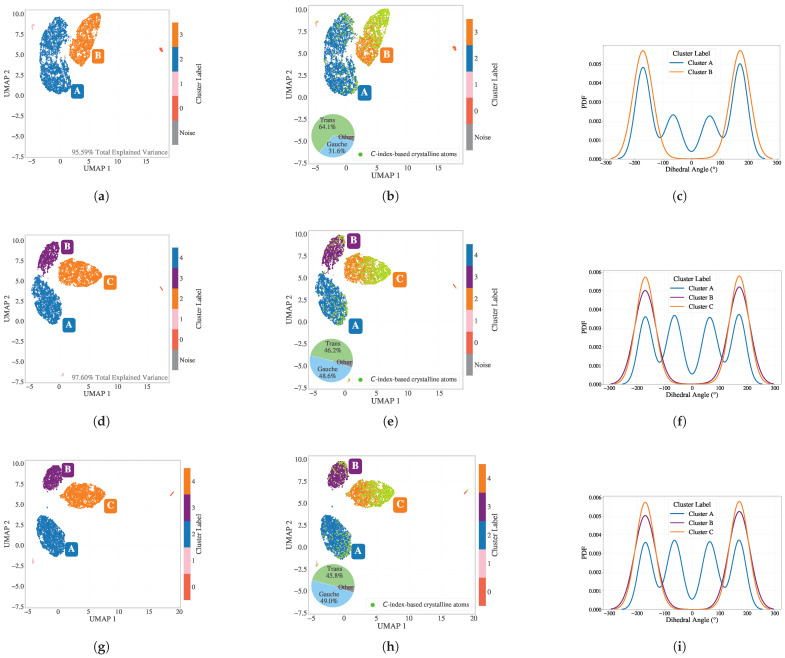
Comparison of SOAP dimensionality reduction pipelines at $t_{mid}$. Panels (**a**–**c**) PCA with 15 components + UMAP + HDBSCAN: (**a**) 2D embedding with 95.59% explained the retained variance; (**b**) colored by $p_{2}$; (**c**) dihedral distribution for different clusters. Dihedral proportions for cluster A (64% trans, 31% gauche) are shown in the inset pie chart of panel (**b**). Panels (**d**–**f**) PCA with 25 components + UMAP + HDBSCAN: (**d**) embedding with 97.60% explained the retained variance; (**e**) colored by $p_{2}$; (**f**) dihedral distribution for different clusters with dihedral proportions (46% trans, 49% gauche) shown in the inset pie chart in panel (**e**). Panels (**g**–**i**) Direct UMAP + HDBSCAN: cleanest separation and consistent dihedral interpretation.

To assess how best to reduce these high-dimensional data, dimensionality reduction was performed at a representative intermediate time step ($t_{mid}$). In Figure 3, three approaches are compared: Figure 3a–c PCA for 15 components followed by UMAP to 2D; Figure 3d–f PCA for 25 components followed by UMAP; Figure 3g–i direct UMAP without PCA. For PCA-based reductions, the total retained variance was 95.59% with 15 components and 97.60% with 25 components (shown in Figure 3a and Figure 3d, respectively). The retention of the variance for the PCA projections is summarized in Appendix A.2 Figure A2. In Figure 3a, the 2D embedding after PCA(15)+UMAP is shown. When colored by $p_{2}$ values (Figure 3b), two major clusters emerge, albeit with considerable noise within the crystalline cluster, while $p_{2}$ shows noncrystal particles as well. Interestingly, when plotted by the dihedral backbone conformation (Figure 3c), the entire orange cluster consists of trans conformations, whereas the blue cluster includes both trans and gauche conformations (with approximately 64% trans and 31% gauche).

Figure 3d shows PCA(25)+UMAP, where the embedding reveals three clusters. In Figure 3e, classification $p_{2}$ highlights the crystalline cluster, which is now more distinct from the rest. The dihedral coloring (Figure 3f) reveals that both the orange and purple clusters correspond to trans conformations, while the blue cluster retains mixed conformations, with around 46% trans and 49% gauche.

Figure 3g presents the direct UMAP result, which shows even greater separation among the clusters. Again, Figure 3h highlights the $p_{2}$-classified crystalline particles concentrated in the orange cluster. Cluster A includes 46% trans and 49% gauche dihedral configurations. Figure 3i confirms the trans and gauche conformation separation. Given cleaner separation and the potential to retain subtle features, the direct UMAP pipeline was selected for SOAP dimensionality reduction. Since the resulting UMAP embeddings showed well-separated clusters, it was not necessary to perform a detailed hyperparameter benchmark for HDBSCAN in this case.

Pie charts were added next to cluster A in the embeddings to display the proportion of trans versus gauche conformations. Furthermore, small peripheral clusters across all UMAP embeddings were found to correspond to chain-end particles or those immediately adjacent to them.

All analyses were performed on per-particle SOAP descriptors computed in the quiescent state without alignment or centering, ensuring consistent treatment of local chain geometry across the system. Researchers adopting different SOAP parameterizations that lead to even higher dimensional feature spaces are advised to repeat this PCA+UMAP comparison. UMAP performance may be degraded in extremely high dimensions [84], and overaggressive PCA may ignore informative variance.

**Figure 4:**
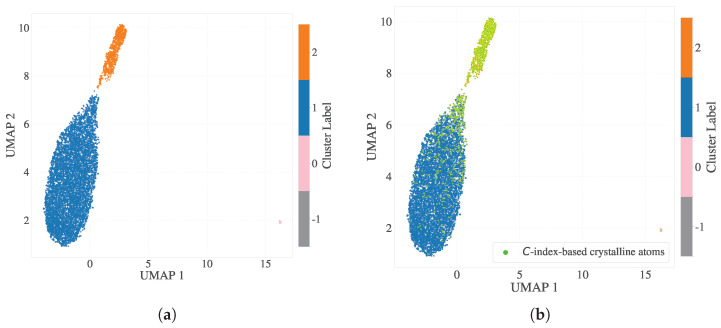
Dimensionality reduction and clustering of SOAP descriptors incorporating 20 consecutive time steps around $t_{mid}$. (**a**) Two-dimensional UMAP embedding of PCA(25)-reduced SOAP vectors (6480 dimensions per particle) with HDBSCAN clustering. (**b**) Particles are colored based on $p_{2}$-based crystallinity classification. The crystalline cluster closely aligns with the cluster identified by HDBSCAN, as the yellow–green points correspond with the orange points identified through clustering in the 2D embedding. However, instantaneous dihedral information was not preserved in this analysis due to the concatenation of temporal data.

To investigate the temporal stability of SOAP-based classifications, we extended each particle’s descriptor by concatenating SOAP vectors across 20 consecutive time steps centered around $t_{\mathrm{mid}}$, ranging from $t_{\mathrm{mid}}-10\Delta t$ to $t_{\mathrm{mid}}+10\Delta t$. This resulted in high-dimensional fingerprints of size 324×20=6480 per particle. Dimensionality reduction was performed using PCA (retaining 25 components), followed by UMAP to embed the data into two dimensions before being subsequently clustered using HDBSCAN (Figure 4a). The resulting clusters aligned closely with those classified as crystalline by the scalar parameter ($p_{2}$; Figure 4b), although the stacked temporal representation precludes the analysis of instantaneous conformational states such as dihedrals.

A similar strategy was applied in our previous study for bond orientational order (BOO) descriptors using 5, 10, and 20 time-step windows, where longer temporal windows led to progressively improved separability between phases [32]. This finding echoes the observations of Adorf et al. [42], who demonstrated that incorporating time into high-dimensional spherical harmonic descriptors significantly improves clustering resolution. However, despite its benefits, we do not adopt temporally stacked descriptors in the remainder of this study, as the goal here is to study OPs that achieve high performance on the same-time structures so that we can move towards the creation of better and more parsimonious models of high-temporal-resolution dynamics of structures; e.g., nucleation and surface growth. Simple concatenation of features over time and use of opaque models would not help in this regard.

### 3.2. Directional Entropy Bands: Resolving Local Configurational Order

#### 3.2.1. Manifold Learning and Comparison of Band-Averaged and Scalar Entropy

**Figure 5:**
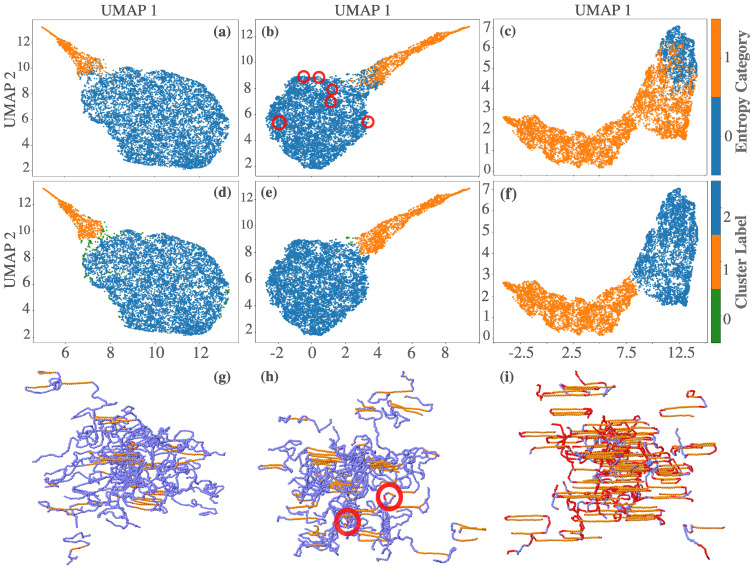
UMAP analysis of shell-based entropy vectors (*entropy bands*) combined with enthalpy values for three key crystallization stages. Panels (**a**–**c**) UMAP embeddings for $t_{tr}$, $t_{mid}$, and $t_{ss}$, respectively, colored by scalar entropy. Red circles in Panel (**b**) highlight discrepancies where scalar entropy classifies particles as crystalline, yet their embedding position suggests otherwise. Panels (**d**–**f**) HDBSCAN clustering applied to the UMAP embeddings of entropy bands, identifying two major structural populations. Panels (**g**–**i**) Structural reconstructions of the clusters at $t_{tr}$, $t_{mid}$, and $t_{ss}$, respectively, displayed using unwrapped atomic-particle coordinates. Misclassified particles from Panel (**b**) appear in folded or bridged segments in Panel (**h**), demonstrating the improved sensitivity of entropy bands in capturing configurational complexity beyond scalar entropy metrics. In Panel (**i**), red particles represent disordered regions—primarily folded segments—that are incorrectly classified as crystalline by scalar entropy (Panel (**c**)) but correctly identified as amorphous by scalar entropy bands. Orange particles correspond to atoms consistently classified as crystalline by both methods.

**Figure A1:**
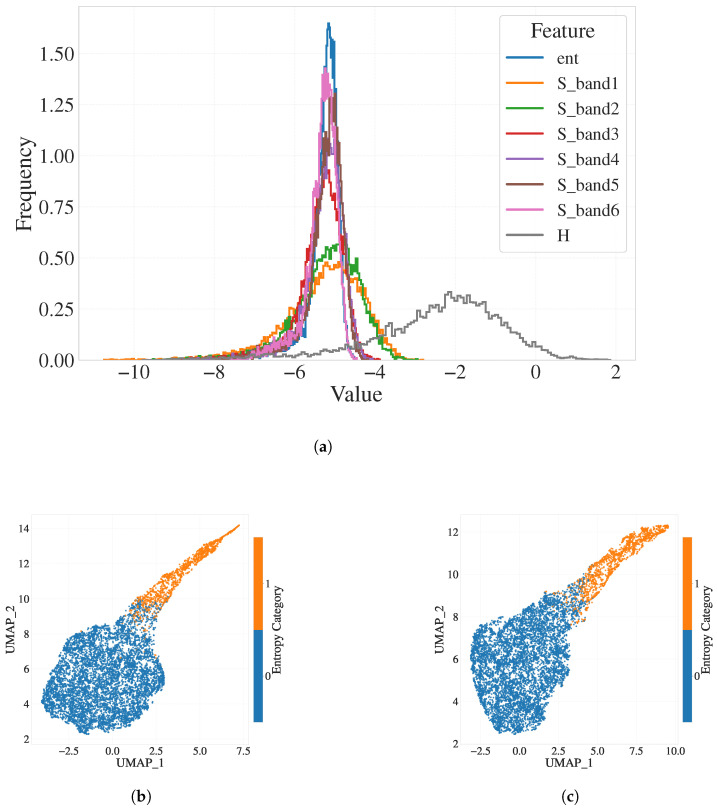
(**a**) Histograms of raw features at an early crystallization stage: scalar entropy (*ent*), six entropy-band components (*$S_{band1}$ to $S_{band6}$*), and enthalpy (*H*). Notice the skew in several bands and in enthalpy. (**b**,**c**) 2D UMAP embeddings (for the transitional snapshot $t_{tr}$), colored by the discrete entropy category. (**b**) after standard scaling; (**c**) after Yeo–Johnson power transform. While the power transform slightly reduces skew in the latent space, the overall manifold structure and cluster separation remain very similar, so we proceed with standard scaling for simplicity.

**Figure A2:**
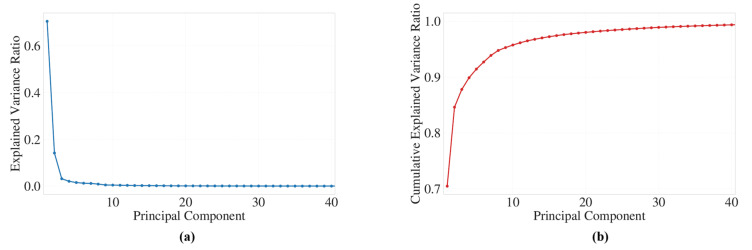
(**a**) Scree plot showing the individual variance contributions of principal components, illustrating the point of diminishing returns beyond the first 15 components. (**b**) Cumulative explained variance plot indicating where more than 95% of the total variance is captured, supporting the selection of the first 15 components as a practical cutoff for our analyses. These plots validate our approach to reducing dimensionality in the SOAP descriptor space.

To investigate whether entropy bands provide enhanced structural resolution over scalar entropy, we applied UMAP to the entropy band vectors combined with local enthalpy (see Appendix A.1 Figure A1) for a comparison of feature scaling methods, including skewness correction of thermodynamic-like parameters prior to dimensionality reduction). Figure 5 presents the resulting embeddings in three representative crystallization stages: transitional ($t_{tr}$), intermediate ($t_{mid}$), and steady state ($t_{ss}$). Figure 5a–c show the UMAP projections colored by the scalar entropy threshold (${\bar{S}}_{i}^{*}=-5.8$ in dimensionless LJ units). These embeddings highlight the absence of a sharp phase separation, especially as crystallization proceeds (Figure 5c). Interestingly, many atoms classified as crystalline by scalar entropy appear in amorphous regions of the entropy band manifold—most notably, in folded or bridged regions—suggesting that scalar entropy fails to capture key configurational nuances. The red circles in Figure 5b mark such discrepancies at $t_{mid}$.

To further investigate these structural groupings, we applied HDBSCAN to the UMAP representations (Figure 5d–f). The resulting clusters broadly align with amorphous and crystalline regions, and boundary overlaps diminish as crystallization matures. Compared to scalar entropy, these clusters offer a more refined partitioning of the phase space, particularly in regions involving interfacial or partially ordered configurations.

The spatial distribution of these clusters is visualized in Figure 5g–i, showing particle-level reconstructions at $t_{tr}$, $t_{mid}$, and $t_{ss}$, respectively. In the early stages (Figure 5g), the differences in clustering are minimal. By $t_{mid}$ (Figure 5h), the entropy bands correctly classify folded-chain atoms misidentified by scalar entropy. At steady state (Figure 5i), the entropy bands robustly distinguish the folded, bridged, and chain-end segments, underscoring their improved sensitivity to configurational heterogeneity.

While ${\bar{S}}_{i}^{\left(b\right)}$ represents a spatial average within concentric radial shells and ${\bar{S}}_{i}$ captures total local entropy, the two differ in their ability to resolve phase interfaces. Band-averaged entropy demonstrates improved separability between amorphous and crystalline regions, as evidenced by UMAP manifolds and HDBSCAN clustering results. However, this separation remains incomplete: the manifold does not exhibit fully disjoined clusters, and HDBSCAN classification near phase boundaries is sensitive to hyperparameters and density fluctuations. These ambiguities particularly affect interfacial or partially ordered atoms, where scalar descriptors—even band-averaged ones—struggle to provide consistent phase assignments.

To address this limitation, we now turn to the full directional entropy vector (${\vec{S}}_{i}^{\left(b\right)}$), which retains first-order angular information about the local entropy field. This vectorized representation is sensitive to configurational anisotropy and directional gradients, key signatures of folded structures, interfacial asymmetries, and structural motifs that are often overlooked by scalar metrics. In the next subsection, we examine whether ${\vec{S}}_{i}^{\left(b\right)}$ offers improved phase discrimination and interfacial resolution, particularly compared to the crystallinity index.

#### 3.2.2. Comparison of Directional Entropy Bands (${\vec{S}}_{i}^{\left(b\right)}$) with the Crystallinity Index (C-index) and Phase Boundaries

To evaluate the spatial and phase-resolution capacity of the directional entropy bands, we applied UMAP to feature sets that comprise the full directional entropy vector (${\vec{S}}_{i}^{\left(b\right)}$). These features include the band-averaged entropy, directional projections (${\bar{S}}_{ix}^{\left(b\right)},{\bar{S}}_{iy}^{\left(b\right)},{\bar{S}}_{iz}^{\left(b\right)}$), the normalized angular moment (${\bar{S}}_{im}^{\left(b\right)}$), and the surface-weighted entropy gradient ($f_{i}$). Full details of the structure of the feature set are provided in Section 2.2.3.

**Figure 6:**
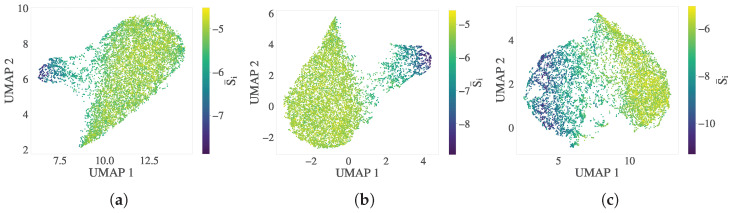
UMAP embeddings of the directional entropy vector (${\vec{S}}_{i}^{\left(b\right)}$) at three crystallization stages ((**a**) $t_{tr}$, (**b**) $t_{mid}$, and (**c**) $t_{ss}$), colored by scalar entropy (${\bar{S}}_{i}$). The embeddings reveal smooth phase separation, reflecting the influence of interfacial and directional features.

Figure 6 shows the embeddings of ${\vec{S}}_{i}^{\left(b\right)}$ in the same three key crystallization stages: transitional ($t_{tr}$), intermediate ($t_{mid}$), and steady state ($t_{ss}$). Figure 6a–c display UMAP projections colored by scalar entropy (${\bar{S}}_{i}$). These embeddings reveal smooth—although not sharply delineated—phase separation, particularly at later crystallization stages (Figure 6c), where increasing interfacial heterogeneity becomes prominent. This outcome is intentional, as the set of characteristics includes the surface-weighted entropy gradient ($f_{i}$), which improves sensitivity to transitional and boundary atoms. Excluding $f_{i}$ leads to sharper melt–crystal separation in the manifold but at the expense of interfacial resolution, which is essential to characterize crystal growth dynamics.

The surface-weighted entropy gradient parameter ($f_{i}$) plays a critical role in enhancing sensitivity to interfacial structure. In this study, we selected $\sigma_{s}=1.5$ for $f_{i}$ as a representative value that balances resolution and stability at different stages of crystallization. Qualitatively similar results are obtained for $\sigma_{s}$ values in the range of 0.1 to 2.0, with minor changes in the position of the inferred interface. Specifically, increasing $\sigma_{s}$ tends to bias the interfacial boundary slightly towards the crystalline side, reflecting a stronger surface weighting. This choice ultimately depends on the desired trade-off between strict geometric separation and the inclusion of interfacial disorder. A comparison of UMAP embeddings and C-index divergence maps across the range of $\sigma_{s}\in \left[0.1,2.0\right]$ is provided in Appendix A.4 Figure A4.

In general, directional components and $f_{i}$, together, introduce sufficient angular and boundary sensitivity to delineate physically meaningful subpopulations–corresponding to melt, crystalline cores, and structurally complex interfacial regions–that are not easily distinguishable using scalar entropy alone or any other order parameter.

**Figure 7:**
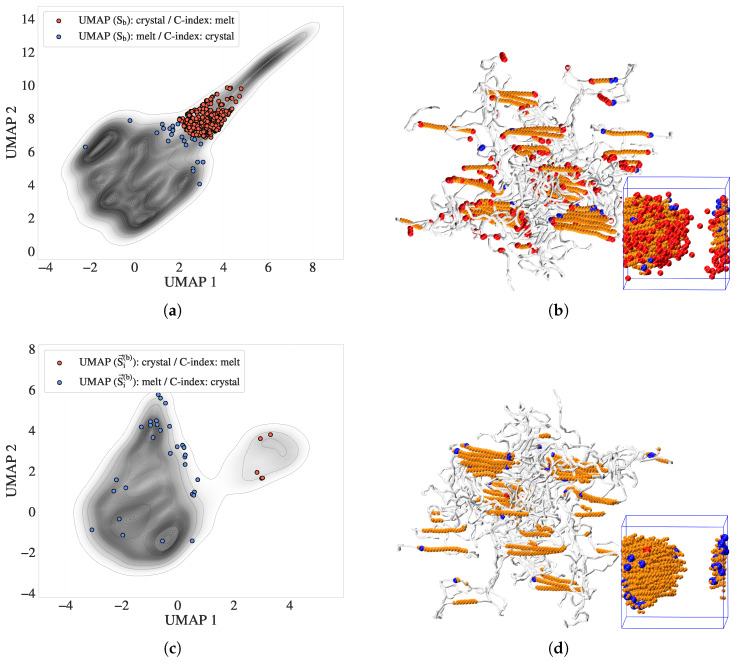
Comparison of scalar entropy bands and directional entropy vectors with the crystallinity index (C-index) at $t_{\mathrm{mid}}$. (**a**) Contour map of scalar entropy bands (${\bar{S}}_{i}^{\left(b\right)}$), with discordant atoms highlighted: red atoms are classified as crystalline by entropy bands but amorphous by the C-index, and vice versa for blue atoms. (**b**) VMD snapshot of the same atoms from (**a**), showing their spatial distribution. Orange atoms represent crystalline regions where the two methods agree; red and blue atoms lie predominantly at interfaces and folded chain segments. (**c**) UMAP embedding of the directional entropy vector (${\vec{S}}_{i}^{\left(b\right)}$), showing reduced phase disagreement relative to (**a**). (**d**) VMD visualization of (**c**), confirming improved agreement, with local structure and enhanced resolution of interfacial atoms.

To further assess the effectiveness of ${\vec{S}}_{i}^{\left(b\right)}$ in resolving local phase identity, we focus on the intermediate crystallization stage ($t_{\mathrm{mid}}$), where phase boundaries are the most structurally uncertain. Figure 7 compares the directional entropy vectors with the C-index in four panels.

Figure 7a presents a map of the scalar entropy band (${\bar{S}}_{i}^{\left(b\right)}$), with atoms highlighted in red or blue to indicate disagreement with the C-index: red atoms are labeled as crystalline by the entropy bands but amorphous by the C-index, whereas blue atoms show the opposite discrepancy. Figure 7b maps these discordant atoms onto a VMD-rendered [87] snapshot of the polymer configuration. Orange atoms correspond to regions where both descriptors agree on crystallinity, whereas red and blue atoms lie predominantly at interfaces or folded chain regions. Figure 7c shows a contour plot of the manifold built from ${\vec{S}}_{i}^{\left(b\right)}$, colored by discordance with the C-index. Compared with Figure 7a, the number of conflicting atoms is significantly reduced. Figure 7d displays improved consistency between DEB predictions and local structure from C-index results, with fewer discordant atoms (red/blue) on the surface of the crystalline regions. These comparisons illustrate that directional entropy vectors align better with the physical morphology and reduce classification ambiguity at phase boundaries.

To further validate the ability of the directional entropy bands to resolve the interfacial structure, we adopted a geometric surface reference (’silver standard’) derived from alpha shapes constructed on the C-index defined crystalline cluster. This approach identifies atoms located on the geometric boundary of the cluster at $t_{\mathrm{mid}}$, independent of entropy-based criteria.

**Figure A3:**
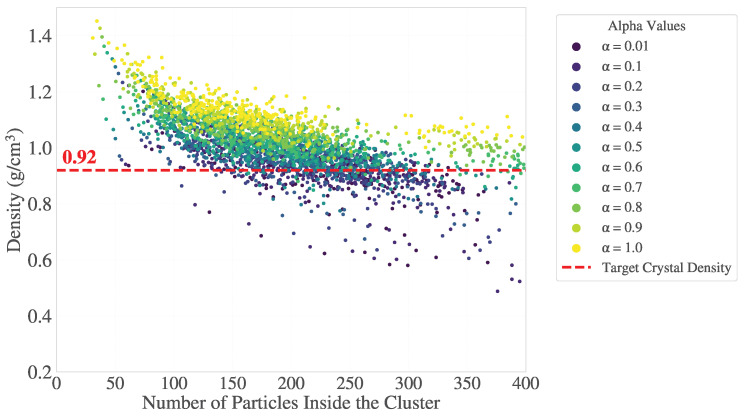
Density of alpha-shaped crystalline clusters as a function of the number of enclosed particles, evaluated across multiple alpha values throughout the growth trajectory. Each point corresponds to one cluster at a given time step and α. The red dashed line indicates the equilibrium crystalline density (0.92 g/cm^3^), obtained from an independently equilibrated bulk simulation using the same force field and model.

**Figure A4:**
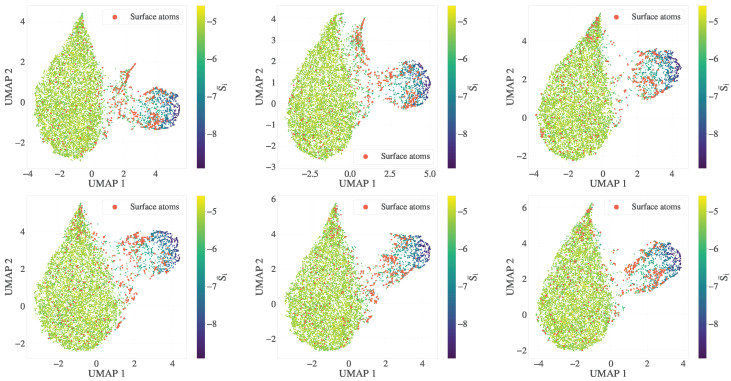
UMAP embeddings of ${\vec{S}}_{i}^{\left(b\right)}$ at $t_{\mathrm{mid}}$ for six values of the entropy gradient smoothing parameter ($\sigma_{s}$): (top row, left to right) $\sigma_{s}=0.1$, 0.2, and 0.5; (Bottom row, left to right) $\sigma_{s}=1.0$, 1.5, and 2.0. Red dots mark atoms located on the alpha-shape surface (α=0.7). A lower $\sigma_{s}$ yields sharper separation but may underdetect soft interfaces; a higher $\sigma_{s}$ smooths directional gradients and broadens the transitional region. The selected value of $\sigma_{s}=1.0$ achieves balanced sensitivity and stability.

We examined the use of alpha shapes to define geometric boundaries on the C-index defined crystalline cluster. Based on Appendix A.3 Figure A3, a suitable range for the alpha parameter is α∈[0.3,0.7], where the balance between over-smoothing (too small of an α) and a sharper surface (too large of an α) is optimal. For the results shown in this subsection, we selected α=0.7 to include a larger number of atoms on the crystal–melt boundary as surface atoms. This choice facilitates both a richer visual interpretation of interfacial behavior in UMAP space and a broader set of labels for subsequent classification tasks.

**Figure 8:**
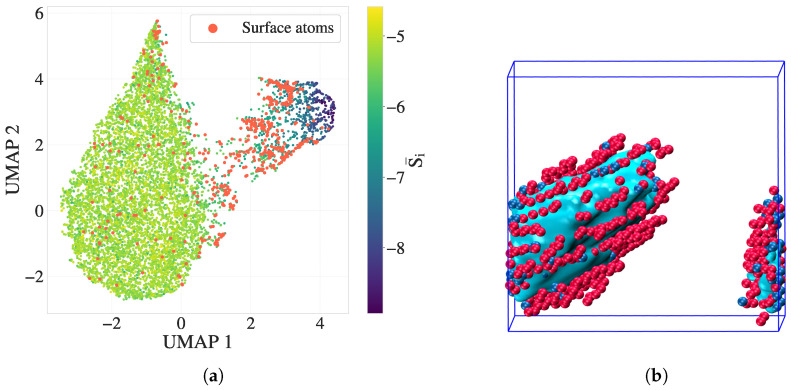
Surface atom identification using the C-index alpha shape (silver standard) and directional entropy bands at $t_{\mathrm{mid}}$. (**a**) UMAP embedding of ${\vec{S}}_{i}^{\left(b\right)}$, with surface atoms highlighted in red. These atoms form a transitional group between melt-like and crystalline clusters. (**b**) VMD snapshot of the crystalline cluster (cyan), overlaid with silver-standard surface atoms (red) and DEB-inferred surface atoms (blue). The strong correspondence illustrates the interface-resolving capability of directional entropy bands.

Figure 8 presents a complementary comparison in both feature space and real space. Figure 8a shows the UMAP embedding of ${\vec{S}}_{i}^{\left(b\right)}$, where the silver standard surface atoms are colored red. These atoms form a distinct transitional group located between melt-like and crystalline clusters in the feature space, consistent with their physical interfacial role. In Figure 8b, a VMD rendering of the crystalline cluster (cyan) is displayed, with overlaid red atoms denoting the alpha shape surface and blue atoms denoting additional DEB-inferred surface atoms to the alpha shape. The DEB-identified surface population closely matches or slightly extends the alpha-shaped boundary, suggesting that it captures both geometric and entropic signatures of the crystal–melt interface.

This correspondence highlights the potential of directional entropy bands to encode interfacial structure with high fidelity, capturing both geometric and entropic signatures of surface atoms. These findings motivate a deeper investigation into whether such features can be leveraged in supervised modeling tasks to systematically identify surface environments.

### 3.3. Model Explanation via Supervised Classification

#### 3.3.1. Motivation and Approach

To explore further the structural information encoded in directional entropy bands, we adopt a supervised classification approach, not to demonstrate the superiority of any particular ML model but to assess whether entropy-based features alone can reproduce known phase assignments and provide interpretable insights into interfacial structure. As target labels, we use the C-index derived in our previous work [32], which combines clustering results from a broad set of conventional order parameters.

Our objective is to determine whether the features of the directional entropy band, derived solely from differential entropy estimates, are sufficiently expressive to learn phase distinctions and surface localization in a physically interpretable way. In particular, our aim is (i) to fit supervised models to establish the predictive power of entropy bands without relying on geometric or symmetry-based descriptors, (ii) to examine label uncertainty near interfacial regions to assess robustness of entropy-based boundaries, and (iii) to identify the most informative subset of components of the entropy band by analyzing the importance of characteristics.

This framework allows us to assess the potential of entropy-derived descriptors to serve as early, physically grounded indicators of interfacial structure, complementing or even anticipating more complex symmetry-based features. In particular, in our previous work [32], we showed that scalar entropy outperforms traditional order parameters in detecting early stage crystallization in entangled polymer chains. Building on that result, the present framework investigates whether directional entropy band features, enhanced with interfacial sensitivity, can improve both interpretation and predictive performance in resolving phase boundaries and surface environments.

#### 3.3.2. Data Preparation

The supervised classification task used band descriptors of directional entropy, including band-averaged entropy (${\bar{S}}_{i}^{\left(b\right)}$), directional projections (${\bar{S}}_{ix}^{\left(b\right)}$, ${\bar{S}}_{iy}^{\left(b\right)}$, and ${\bar{S}}_{iz}^{\left(b\right)}$), max-based gradient estimation (${\bar{S}}_{im}^{\left(b\right)}$), and the surface-weighted entropy gradient ($f_{i}$). All continuous features were standardized to zero mean and unit variance for the random forest model. The dataset was divided into training subsets (80%) and testing subsets (20%) using stratified sampling to maintain class balance.

#### 3.3.3. Model Selection and Training

We evaluated both a Random Forest (RF) classifier and a Logistic Regression (LogReg) model to assess the separability of surface atoms based solely on entropy-derived descriptors. The RF model, known for capturing nonlinear feature interactions and robustness to colinearity, was used as a high-capacity baseline. The hyperparameters (number of estimators and maximum tree depth) were tuned through five-fold stratified cross-validation using grid search to maximize the mean area under the receiver operating characteristic curve (AUC).

To complement this, we also trained a logistic regression model, which provides a linear and additive mapping between input features and predicted probabilities. This model allows for direct interpretation of the feature weights, making it especially suited to identifying combinations of entropy band components that align with a physically meaningful interfacial structure. Our goal here is not to optimize predictive performance per se but to evaluate whether a linear combination of entropy-based features is sufficient to distinguish surface environments, a valuable step toward building a mechanistic understanding of nucleation and growth processes.

The surface atom labels used for classification were generated using alpha shapes constructed in the C-index defined crystalline cluster, as described in Section 3.2.2. Although alpha-shape surfaces are geometric proxies and not exact thermodynamic boundaries, they serve as a useful silver standard for assessing the extent to which directional entropy bands capture interfacial structure.

#### 3.3.4. Performance Evaluation: ROC Analysis

**Figure 9:**
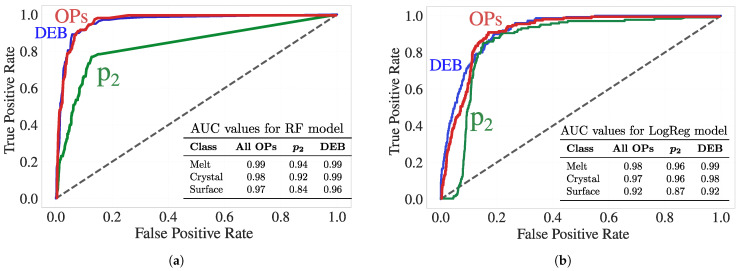
ROC curves for surface classification using (**a**) RF and (**b**) LogReg models trained on three feature sets: $p_{2}$, the set of conventional scalar OPs (excluding entropy), and DEB. Only the surface-class ROC curves are shown for clarity. AUC values for melt, crystal, and surface classes are reported in the inset tables. Despite the silver truth labels being derived from the full OP set (including entropy), the DEB features alone exhibit competitive or superior surface-prediction performance.

Figure 9 presents the ROC curves for the surface class using both the RF and LogReg models trained on three sets of characteristics: p2, the set of conventional scalar OPs (excluding entropy), and the proposed DEB descriptors. Despite the silver-standard ground-truth labels (i.e., the C-index clusters) being derived using the OPs set (including scalar entropy), DEB descriptors alone achieve comparable or even superior predictive performance. For surface classification, DEB reaches AUCs of 0.92 (LogReg) and 0.96 (RF), outperforming $p_{2}$ (0.87 and 0.84) and closely matching the OP group (0.92 and 0.97), which aggregates more variables.

These results underscore a key contribution of this work: DEB descriptors, built solely from differential entropy without relying on high-order symmetry terms, are not only physically interpretable but also highly predictive of interfacial environments. The fact that DEB matches the performance of a broader OP set, even though the ground-truth clustering was derived from those OPs, highlights its robustness. Furthermore, in light of our previous findings that entropy is a sensitive early-stage indicator of nucleation, these entropy-based features may offer a more timely and mechanistically meaningful route toward understanding crystallization front propagation and interfacial dynamics in polymeric systems.

#### 3.3.5. Uncertainty of Cluster_Label

To quantify the uncertainty of the label in interfacial regions, we manually inspected 600 atoms selected near the boundaries of the UMAP cluster (surface atoms) in three representative time steps (200 atoms per time step). Approximately 10 atoms (∼1.7%) were clearly mislabeled, while more than 100 atoms (∼17%) exhibited ambiguous labeling due to their proximity to phase interfaces. Conservatively, we adopt an estimate of uncertainty of ∼ 2% for clearly mislabeled atoms, acknowledging a potential upper bound of ∼18% if ambiguous cases are considered. This manually estimated uncertainty provides a practical ceiling for the classification of AUCs, beyond which additional predictive gains may reflect overfitting to label noise rather than improved physical resolution of the interfacial structure.

#### 3.3.6. Forward Feature Selection and Model Interpretation

Using both a linear logistic regression model and nonlinear classifiers (RF and XGBoost), we applied forward feature selection (FFS) with five-fold stratified cross-validation to identify key directional entropy-band descriptors for surface atom classification. The descriptors were added one at a time until the AUC of the test set plateaued or approached the noise floor determined by manual inspection (Section 3.3.5).

**Figure 10:**
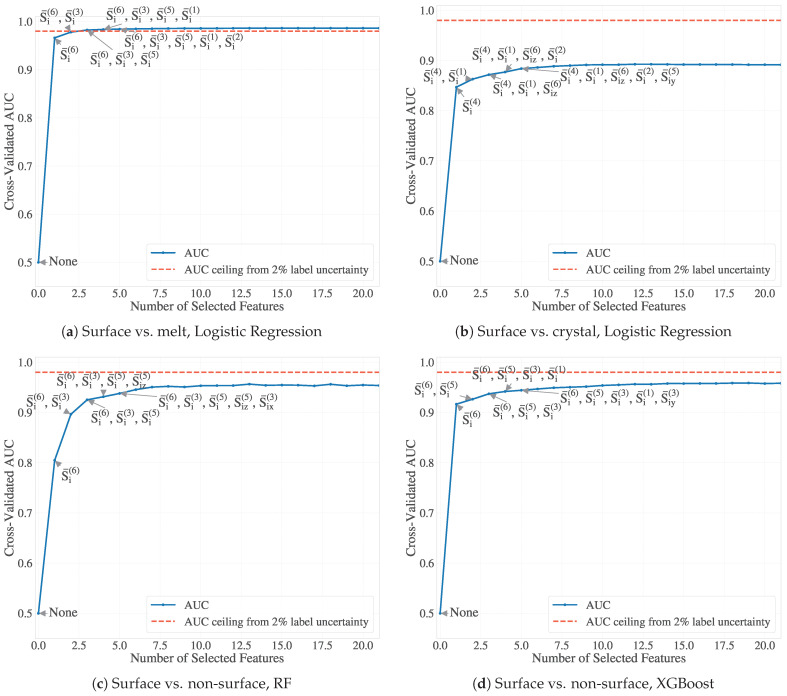
Forward feature selection (FFS) results using ROC-AUC as the performance metric. Classifier performance saturates after only 3–5 directional entropy-band features. XGBoost and RF reach AUC≈0.955 for surface vs. non-surface classification, while logistic regression plateaus at ≈0.985 (surface vs. melt) and ≈0.89 (surface vs. crystal). Dashed red lines mark the practical AUC ceiling (∼0.98–0.99) set by ∼2% labeling uncertainty.

As shown in Figure 10, the performance of the classifier was rapidly saturated, usually after incorporating only three to five directional features of the entropy band. For example, the XGBoost and RF models both reached an AUC of 0.955 for the surface vs. non-surface (including both melt and crystal) classification, while logistic regression plateaued at 0.985 for surface vs. melt and 0.89 for surface vs. crystal. Given our estimated labeling uncertainty of 1–2%, this corresponds to a practical AUC ceiling of approximately 0.98–0.99, so the inclusion of additional features led to only marginal gains. This plateau behavior indicates potential overfitting to boundary noise and confirms that a small, physically significant subset of entropy-band components suffices for accurate surface classification. Including additional features risks fitting spurious correlations rather than capturing meaningful structure, especially in interfacial regions where labels are intrinsically uncertain due to geometric or thermodynamic ambiguity.

Logistic regression was also applied separately to classify surface atoms against either melt or crystal environments. This decomposition avoids confounding effects in three-class linear models and reflects the inherent physical asymmetry between the two interfacial types. In the main model used herein, constructed with α=0.7 for alpha-shape surfaces and σ=1.5 for ${\vec{f}}_{i}$, we observed that surface–crystal boundaries were harder to distinguish, consistent with tighter packing and reduced entropy gradients near the crystalline domains. These surface atoms are more deeply embedded and less geometrically distinct from the bulk, whereas the surface–melt interface exhibits broader spatial separation and more pronounced configurational contrast.

**Figure A5:**
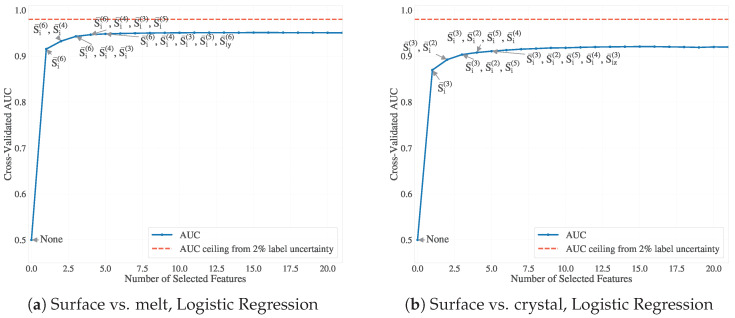
Forward feature selection using DEB descriptors under stricter surface definitions. (**a**) Logistic regression model distinguishing surface from melt atoms; (**b**) logistic regression distinguishing surface from crystalline atoms. These results correspond to interface definitions based on α=0.3 (alpha shape) and σ=0.2 (surface-weighted entropy gradient). While the melt interface becomes harder to resolve under this configuration, surface–crystal classification improves significantly. In all cases, performance saturates after a few DEB features, highlighting their robustness across surface extraction parameters.

To examine whether the observed asymmetry is due to model resolution or label definition rather than intrinsic structural complexity, we repeated the classification using hyperparameters of α=0.3 for the alpha-shaped surface and σ=0.2 for the weighting of the entropy gradient in $f_{i}$. This configuration yields a more spacious cluster and a thinner interfacial layer (i.e., fewer surface atoms). As shown in Appendix A.5 Figure A5, the surface–crystal classification performance improved to AUC > 0.91, suggesting that the earlier difficulty was partially due to label ambiguity in those definitions. However, the modest gain in AUC (approximately three percentage points) implies that learning the surface–crystal boundary remains intrinsically more challenging using linear decision boundaries (here, logistic regression). This refinement also came at the cost of surface melt classification, which decreased to AUC ≃ 0.95, probably due to the reduced spatial distinction when the surface region is narrowly defined with lower values of α.

These results suggest that surface atom classification using DEB descriptors is consistently robust across different interface definitions. The set of DEB components required to reach performance saturation may vary depending on the classifier and hyperparameters, but the overall predictive power remains strong. Importantly, we observe that AUC values plateau after inclusion of only a few DEB features, underscoring their relevance and sufficiency for identifying interfacial atoms in polymeric systems with varying structural resolution.

#### 3.3.7. Diffuse Nature of Polymer Interfaces and the Role of DEB

The joint evidence from (i) uncertainty analysis, (ii) classifier asymmetry, and (iii) α-shape sensitivity supports a coherent picture: polymer crystal–melt interfaces are not razor-thin but occupy a structurally graded zone of apparent thickness that depends on operational definitions. Therefore, the role of DEB is not to enforce an artificially sharp separator but *to parameterize the graded anisotropy* of local environments in a compact, interpretable form. This interpretation aligns with experimental reports of interfacial gradients and enhanced dynamics [58,88,89] and explains why a few DEB components capture most of the predictive signal while additional features mainly chase boundary noise.

To capture nonlinear interactions among entropy-band descriptors, we also trained an XGBoost classifier for surface vs. non-surface (melt + crystal) classification. This model achieved a high AUC (≥0.985) using only three to four features, indicating a strong discriminative power of the directional entropy bands. The SHAP values were computed to interpret these nonlinear decision boundaries, focusing on surface atoms and stratifying results by radial and longitudinal surface geometries. Together, these results confirm that directional entropy bands are physically grounded, compact, and effective variables for surface detection and provide a foundation for future modeling of interfacial dynamics.

#### 3.3.8. Feature Importance and Local Structure Patterns

**Figure 11:**
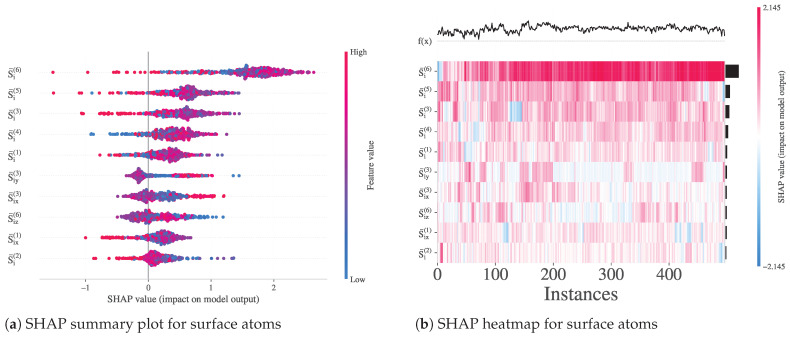
SHAP value analysis for the XGBoost model trained to distinguish surface atoms. (**a**) Summary plot showing the contribution of each feature across atoms labeled as surface (Cluster_Label = 1). (**b**) Heatmap visualization of SHAP values across surface atoms, illustrating consistent patterns in the importance of entropy-band features.

To interpret the trained nonlinear XGBoost classifier for surface detection, we calculated SHAP values over the subset of surface atoms (Cluster_Label = 1). The SHAP summary plot (Figure 11a) highlights that the most influential features are the band-averaged entropies (${\bar{S}}_{i}^{\left(b\right)}$), particularly from the outermost and midrange shells (b=6, 5, and 3), along with directional components such as ${\bar{S}}_{ix}^{\left(3\right)}$ and ${\bar{S}}_{iy}^{\left(3\right)}$. This confirms the central role of band-resolved entropy profiles in characterizing interfacial order. In particular, both positive and negative SHAP values appear across bands, indicating nonmonotonic interactions between local order and surface classification. The heatmap (Figure 11b) further reveals structured, non-random patterns in SHAP values across atoms, with feature contributions varying coherently between instances. These results demonstrate that directional entropy bands capture reproducible interfacial features with high discriminative value, validating their use in subsequent modeling of surface and transition dynamics.

#### 3.3.9. Summary of Insights from DEB

DEB furnishes a compact, physically grounded coordinate system for interfacial structure: a few band-resolved entropy components are sufficient (i) to trace the graded melt–surface–core manifold, (ii) to separate the surface from the melt phase at near noise-ceiling accuracy, and (iii) to approach the intrinsically harder surface–crystal boundary without overfitting. The residual performance gap is consistent with experimentally documented diffuse interfaces and with the sensitivity of any classifier to operational labels at the boundary. These observations motivate the temporal and mechanistic studies outlined in Section 4, where DEB will be used as a low-dimensional state variable for surface kinetics, early nucleation signaling, and interfacial free-energy modeling.

## 4. Conclusions

We have introduced *directional entropy bands* (DEB): shell-resolved, angularly projected extensions of local entropy that augment scalar entropy with first-order directional moments and radial entropy gradients. In equilibrium polymer crystallization simulations, these descriptors (24–30D) produce a continuous melt–surface–core manifold in UMAP space and separate interfacial atoms with fidelity higher than conventional order parameters (scalar entropy, $q_{6}$, $p_{2}$, and SOAP) at single-snapshot resolution. Forward feature selection and binary classification demonstrate that (i) surface vs. melt discrimination attains near-noise-ceiling AUC (∼0.98) with three band-averaged entropy features; (ii) surface vs. crystal separation is intrinsically harder (plateau AUC ∼0.90), reflecting structural proximity of the surface and crystalline core; and (iii) a nonlinear model (XGBoost) surface vs. non-surface reaches an AUC ≳0.955 using only three to four DEB features, confirming that added performance arises from feature interactions rather than large feature sets. SHAP analysis restricted to surface atoms shows that the dominant contributions arise from the outer and mid shells (b=6,5,3) with secondary directional components, validating that the anisotropic interfacial order is captured by low-order angular entropy projections without recourse to high-dimensional spectrum fingerprints. The ability of a compact family of entropy-centric, physically interpretable variables to match or exceed rich fingerprints for interfacial detection underscores that the directional organization of local entropy is a primary driver of the crystallization surface signal.

### Future Directions

The present work is a first step toward a surface-centric thermodynamic–kinetic modeling framework. Extensions include (1) **temporal predictions**—contrasting same-time detection with lagged prediction to isolate features whose signal precedes growth rather than merely correlating with established surfaces and forecasting future surface formation (melt → surface attachment) to identify components with predictive (possibly causal) leverage; (2) **early nucleation signaling**—quantifying advance warning times of DEB vs. conventional OPs for emergent crystalline regions; (3) **surface taxonomy**—unsupervised and supervised classification of anisotropic surface classes (facet orientations and fold/edge motifs) using DEB subsets; and (4) **coarse thermodynamic modeling**—fitting effective interfacial free-energy or Gibbs-like functionals in reduced DEB space across chain lengths, thermal protocols, and flow conditions. These directions aim to translate the high discriminative power and ease of interpretation of directional entropy bands into predictive, mechanistic models of polymer crystal growth kinetics.

## Data Availability

The data that support the findings of this study are available from the corresponding authors upon reasonable request.

## Supplementary Materials

The following supporting information can be downloaded at: https://www.mdpi.com/article/10.3390/polym17172399/s1, Figure S1: Equilibration and thermodynamic validation (energy, ACF, melting/crystallization temperatures, and density trace); Figure S2: UMAP hyperparameter optimization heatmaps for Euclidean and Manhattan metrics; Figure S3: HDBSCAN hyperparameter sweep and representative cluster assignments; Figure S4: Ensemble classifiers hyperparameter grid searches; Figures S5 and S6: Model comparison bar plots and representative confusion matrices; Figure S7: Higher-molecular-weight (C500) demonstration of DEB embeddings; Code/Data S1: Compressed archive SM_DEB_SOAP.zip containing DEB and SOAP calculations with a sample dataset ($t_{mid}$), Data analysis scripts (UMAP, HDBSCAN, supervised classifiers), and plotting routines (documented in a README.txt). The code is also maintained at our GitHub repository https://github.com/etourani/DEB (accessed on 28 August 2025). Reference [90] is cited in the supplementary materials

## Abbreviations

The following abbreviations are used in this manuscript:

MD	Molecular Dynamics
PE	Polyethylene
UA	United Atom
OP	Order Parameter
DEB	Directional Entropy Bands
UMAP	Uniform Manifold Approximation and Projection
SOAP	Smooth Overlap of Atomic Positions
HDBSCAN	Hierarchical Density-Based Spatial Clustering of Applications with Noise
ROC	Receiver Operating Characteristic
AUC	Area Under the Curve (of the ROC)
SHAP	SHapley Additive exPlanations

## Appendix A

### Appendix A.1. Effect of Skewness Correction on UMAP Embedding

To assess the effect of skewed feature distributions—particularly on thermodynamic-like parameters such as enthalpy, entropy, and entropy bands during the early stages of nucleation—a Yeo–Johnson power transform was tested against standard mean–variance scaling (see Figure A1). The Yeo–Johnson method is appropriate for features with both positive and negative values. It helped reduce skewness; however, its effect on the UMAP manifold structure is minimal. Therefore, the standard scaling was retained for the remainder of the analysis to maintain simplicity and consistency.

### Appendix A.2. PCA for SOAP Descriptors

In the scree plot, (Figure A2a), each point on the y-axis is the percentage of total variance explained by that component alone. We see that once we pass approximately components 10–20, each subsequent principal component captures only a tiny additional fraction of the variance.

In the cumulative explained variance plot (Figure A2b), 15 components already explain more than 95% of the total variance, so taking more than 15 dimensions from the feature space usually offers diminishing returns. However, a detailed study of this is conducted in the main text (Figure 4).

### Appendix A.3. Selection of Alpha for Geometric Surface Definition

To determine an appropriate alpha value for geometric surface detection, we evaluated the density of alpha-shaped crystalline clusters over time using varying values of the alpha-shape parameter (α∈[0.01,1.0]). For each α, the volume of the alpha shape was calculated, and the effective density was estimated using a corrected count of enclosed atoms, normalized by a simulation-specific scaling factor.

Figure A3 shows the resulting density profiles as a function of the number of enclosed particles. At small α values, the alpha shape becomes more permissive, enclosing fewer boundary atoms and void space, systematically leading to lower densities. Based on this analysis, we find that α values in the range of 0.3 to 0.7 yield physically consistent densities near the expected crystalline value for the system under study. For the visualization and classification tasks described in the main text, we selected α=0.7 to capture a larger set of interfacial atoms without excessive overfitting.

### Appendix A.4. UMAP Response to Surface Smoothing Parameter (σ_s_)

To assess the effect of the surface weighting parameter ($\sigma_{s}$) on the directional entropy gradient ($f_{i}$), we generated UMAP embeddings at $t_{\mathrm{mid}}$ using directional entropy vectors (${\vec{S}}_{i}^{\left(b\right)}$) calculated with varying values of $\sigma_{s}$. In all cases, the geometric reference alpha shape (with α=0.7) is used to highlight the surface atoms in red.

As the value of $\sigma_{s}$ increases, the feature transitions in the UMAP space appear smoother, and the surface regions become slightly wider. At lower $\sigma_{s}$ values (e.g., 0.1–0.2), surface detection sharpens but can miss transitional atoms. At higher values (e.g., 1.5 to 2.0), interfacial atoms blend more deeply into the feature space, slightly reducing surface–crystal separation. Based on this comparison, $\sigma_{s}=0.5$, 1.0, or 1.5 offers a balanced resolution of surface structure and interfacial continuity.

### Appendix A.5. Forward Feature Selection Under Alternative Interface Definitions

As shown in Figure A5, these modifications change the relative difficulty of classification between the two interfaces. In Figure A5a, the performance of logistic regression for surface-melt classification decreases slightly (AUC plateau of ∼0.86), probably due to the reduced spatial separation between melt and surface atoms under tighter surface definitions. In contrast, Figure A5b shows improved classification of surface–crystal atoms (AUC > 0.98), supporting the interpretation that the lower AUC in the original model was partially due to the ambiguity of the label. However, the improvement is modest (from ∼0.89 to >0.98), implying that learning the surface–crystal boundary remains intrinsically more challenging using linear decision boundaries (here, logistic regression).

To evaluate the sensitivity of surface classification performance to the choice of interface-defining parameters, we repeated the forward feature selection (FFS) analysis using a tighter alpha shape parameter (α=0.3) and a more localized entropy gradient weighting (σ=0.2) for computation of ${\vec{f}}_{i}$. This configuration produces thinner and more distinct surface layers at the cost of potentially excluding some transitional atoms.

As shown in Figure A5, these changes alter the relative ease of classification between the surface melt and surface–crystal interfaces. In Figure A5a, the logistic regression model classifies surface versus melt atoms with slightly reduced accuracy (AUC plateau of∼0.955), likely due to reduced boundary resolution under the new surface definition. In contrast, Figure A5b shows significantly improved classification of surface–crystal separation (AUC > 0.91), indicating a clearer distinction when the embedded surface sets are slightly excluded from the crystalline atoms using a smaller alpha value. As with previous configurations, the performance saturates after including only a small subset of directional entropy-band components, demonstrating that the DEB representation is robust to surface definition changes and consistently encodes meaningful interfacial information.
