# Supplementary material for: Directional Entropy Bands for Surface Characterization of Polymer Crystallization
Source: Polymers (Basel). 2025 Sep 3;17(17):2399. doi: 10.3390/polym17172399 (PMC12431618; doi:10.3390/polym17172399)
Supplement: Supplementary file 1 [file polymers-17-02399-s001.zip › polymers-3809164-supplementary.pdf]

Supplementary Material for Directional Entropy Bands for Surface Characterization of Polymer Crystallization

Elyar Tourani[1], Brian J. Edwards*[1], and Bamin Khomami†[1]

[1]Materials Research and Innovation Laboratory, Department of Chemical and Biomolecular Engineering, University of Tennessee, Knoxville, TN 37996, USA

September 2, 2025

*Email: bje@utk.edu
†Email: bkhomami@utk.edu

S1 Equilibration protocol and validation of simulation conditions

We verified that the molecular dynamics (MD) conditions and parameters achieve equilibrium before production analyses, and that the chosen temperature window is physically consistent for polyethylene quiescent crystallization under the Siepmann-Karaborni-Smit (SKS) force model.

Thermalization and barostatting. Starting from a randomized initial configuration at 50 K, the system was heated to 550 K over 5 ns in the canonical ensemble (NVT). We then switched to the isothermal–isobaric ensemble (NPT) at 550 K to relax pressure, cell dimensions, and density. Panel (a) of Fig. S1 shows a plot of the total energy versus time. After the initial transient startup period, the curve reaches a statistically stationary regime with small fluctuations about a constant mean, indicating equilibration of total energy.

Chain relaxation. To ensure configurational relaxation of chains (C150), we computed the normalized end-to-end vector autocorrelation function (ACF). As shown in Panel (b), the ACF decays rapidly at early times and then fluctuates around zero, consistent with loss of memory of the initial configuration. Therefore, the equilibration process for the initial configuration was conducted sufficiently long so that the ACF decayed well below statistical noise, i.e., beyond the longest relevant chain relaxation time.

Thermodynamic consistency of $\mathbf{T_m}$ and $\mathbf{T_c}$. To validate the forcefield and simulation setup, we constructed an independent simulation in which an orthorhombic crystal at $T < 200$ K was heated under NPT, and monitored the enthalpic/structural response. As shown in Panel (c), melting occurs at $T_m \approx 396$ K, which is in close agreement with experimental behavior for polyethylene. A complementary cooling protocol from the melt reveals an onset of crystallization in the range $T_c \approx 260$–320 K, consistent with expectations for this model and system size. These checks confirm that the working temperature window in this study (300K) is within the physically relevant crystallization regime.

Quench protocol and density stability. For nucleation and growth studies, the equilibrated melt (in Panels (a) and (b)) was quenched to 300 K under NPT (Panel (d)). The density exhibits the expected increase during nucleation and early crystallization and then approaches a steady-state plateau as growth proceeds. Analysis frames (including the primary "t_{mid}" snapshot used throughout the main article) were selected from the different stages of the density trace.

Taken together—(i) stationary total energy under fixed T, (ii) decay of chain ACF to zero, (iii) force field-consistent T_m and T_c, and (iv) stabilized density under NPT—these diagnostics demonstrate that the MD conditions achieve equilibrium before production sampling and that the simulated thermal protocol is consistent with the expected thermodynamics of a polyethylene system.

S2 ML hyperparameter tuning

S2.1 Dimensionality Reduction, UMAP

To optimize the UMAP hyperparameters for clustering, we carried out a grid search over `n_neighbors` and `min_dist` values, evaluating each combination using silhouette scores obtained from K-Means and GMM clustering. The number of neighbors, `n_neighbors`, was varied between 5 and 100. We found that smaller values (10–20), especially those close to the average neighborhood size of 15, provided clearer separation between phases in the reduced-dimensional space. In contrast, larger values yielded smoother, more continuous embeddings, but the boundaries between clusters became less distinct. For the `min_dist` parameter, we tested values from the set {0, 0.01, 0.1} and observed that smaller values (0 and 0.01) produced more compact and well-defined phase groupings.

We also examined the impact of different distance metrics, comparing Euclidean and Manhattan distances, as these metrics are known to preserve local and global structures differently in high-dimensional datasets [1]. The silhouette scores, summarized in Figure S2, indicate consistent performance patterns across both metrics, with the best-performing configurations highlighted by the red circle. Based on these results, we selected `n_neighbors` = 10 and `min_dist` = 0.0 using the Manhattan metric for all subsequent clustering and visualization tasks. Overall, the K-Means and GMM analyses

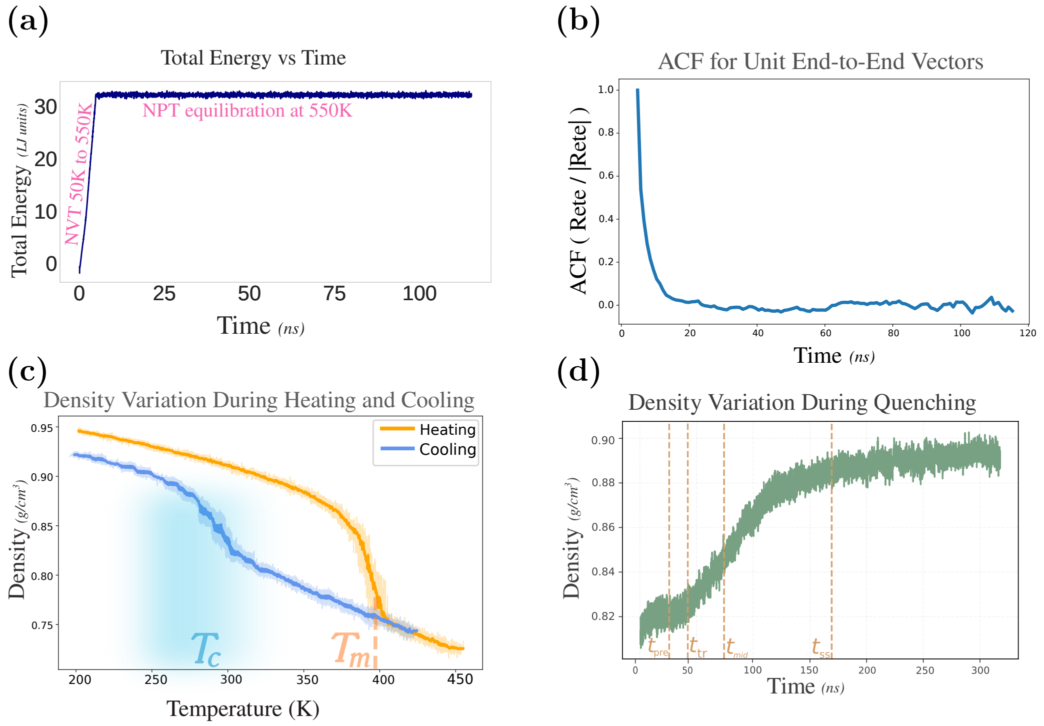

Figure S1: **Equilibration and thermodynamic validation.** (a) Total energy vs. time; after heating from 50 to 550 K over 5 ns (NVT), the system is relaxed at 550 K (NPT) to reach a stationary energy signal. (b) Autocorrelation of the normalized end-to-end vectors for C150 chains, showing rapid decay and subsequent fluctuations about zero, indicating configurational equilibration. (c) Thermal cycling test for the SKS model: an orthorhombic crystal heated from $\sim$ 200 K exhibits melting at $T_m \approx 396$ K; cooling from the melt yields a crystallization window $T_c \approx 260$–320 K. (d) Density vs. time during quench to 300 K (NPT), showing the expected increase during nucleation/crystallization and a subsequent plateau. Analysis frames were selected from the stationary segment (including t_{mid}).

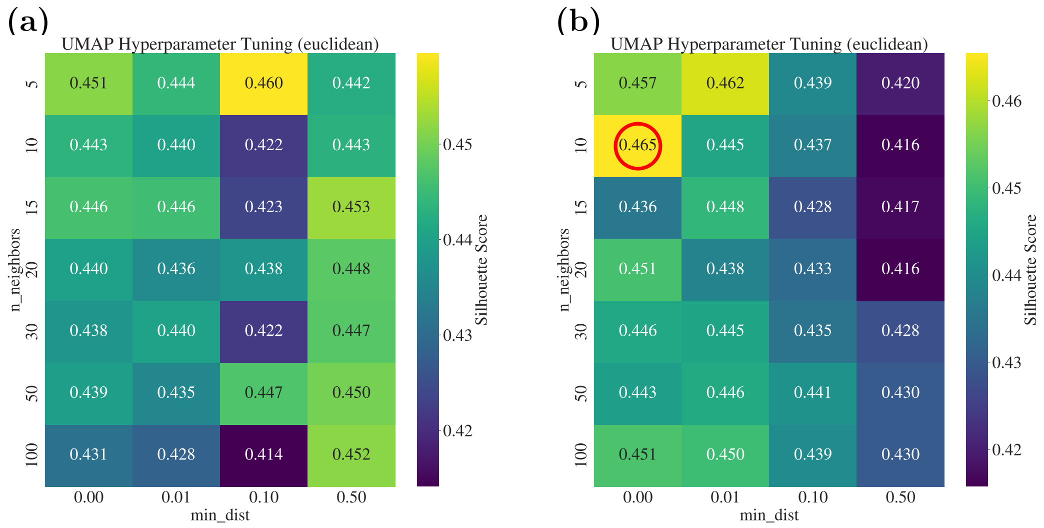

Figure S2: Heatmaps of silhouette scores obtained from UMAP hyperparameter optimization, evaluated using the mean clustering performance from K-Means and GMM across both (a) Euclidean and (b) Manhattan distance metrics. Each panel shows the averaged silhouette score over ten independent runs for varying `n_neighbors` and `min_dist` combinations. The Red circle highlight the hyperparameter settings that achieved the highest silhouette scores.

confirm the stability of clustering outcomes in the 2D UMAP embedding, with closely aligned performance across metrics.

S2.2 HDBSCAN Hyperparameter Optimization

To distinguish between crystalline and amorphous local environments, we applied HDBSCAN to the UMAP-reduced feature space as the primary clustering approach. Prior to tuning HDBSCAN, K-Means and GMM (with a fixed number of clusters, $n = 2$) were used exclusively to determine the optimal UMAP embedding parameters, ensuring maximum phase separation in the reduced-dimensional space. Based on the average silhouette scores from these preliminary tests, the UMAP hyperparameters were fixed at `n_neighbors` = 10 and `min_dist` = 0.0 (see Supplementary Material, Fig. S2).

For HDBSCAN, we explored a wide hyperparameter range relative to system size: min_cluster_size ∈ [0.5%, 5%] of the total atom count and

min_samples ∈ [0.1%, 1.5%] of the system size. Both Euclidean and Manhattan (city-block) distance metrics were evaluated, and while the overall clustering trends were consistent, the precise optimal values for min_cluster_size and min_samples varied slightly between metrics. The leaf size parameter was fixed at 40 (the default).

A grid search was then performed over `min_cluster_size` and `min_samples` for both distance metrics, restricting results to parameter combinations yielding exactly two clusters (crystalline vs. amorphous). The resulting silhouette scores for all tested combinations are summarized in Fig. S3.

Because the DEB feature space projects to a UMAP plane with clear phase separation, the HDBSCAN optima are fairly persistent across the (`min_cluster_size`, `min_samples`) grid, forming broad plateaus of high silhouette score. The small set of points lying between the two clusters in the 2D UMAP space are part of interfacial surface atoms at crystal–melt boundaries. In the supervised modeling section, we confirm this interpretation: those boundary points align with the surface class derived from the crystallinity index (C-index) segmentation, as silver-standard ground truth, and classifiers trained on DEB features learn to identify these surface atoms with strong precision/recall.

S2.3 Hyperparameter Grid Search for Classifiers

We conducted a grid search over tree depth and the number of estimators for both the Random Forest and Gradient Boosting classifiers to evaluate model robustness and to identify appropriate hyperparameter ranges. As shown in Fig. S4, both models achieved consistently high AUC scores ($\gtrsim 0.95$) across the explored parameter space, indicating stable performance with respect to hyperparameter variations.

S2.4 Additional metrics for surface–atom classification

To provide a model comparison, we report accuracy, precision, recall, F1, and AUC for Logistic Regression (two tasks: surface vs. melt and surface vs. crystal), Random Forest (RF), and Gradient Boosting (GB). Figure S5 summarizes the cross-validated performance across models, and Fig. S6 shows representative confusion matrices.

Our objective is not a bake-off between classifiers but to establish that the *Directional Entropy Bands* (DEB) descriptors provide a reliable signal

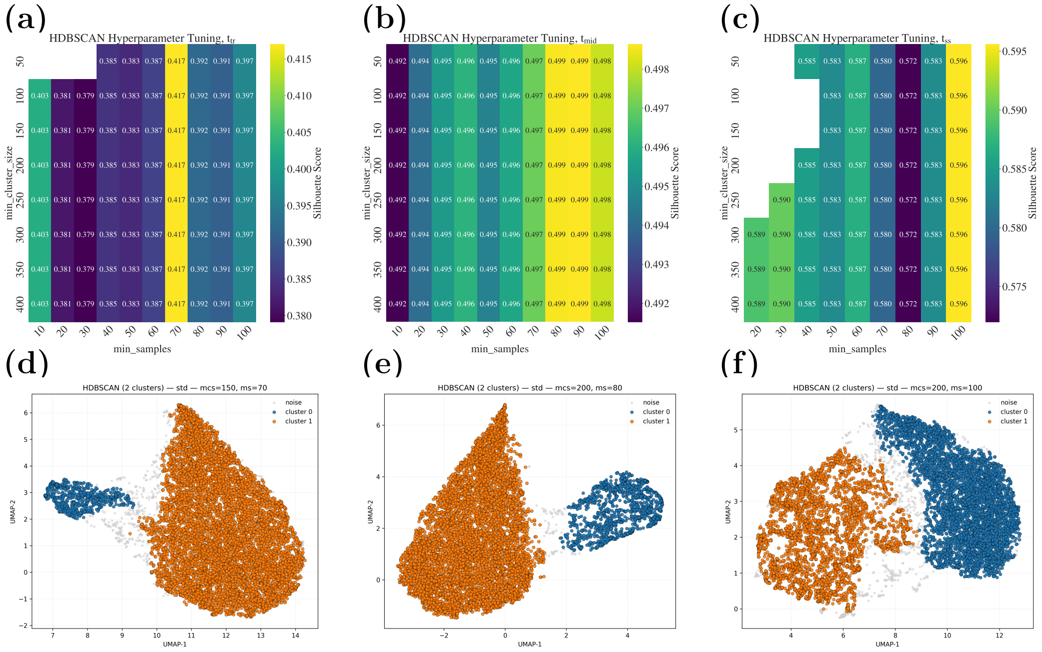

Figure S3: **HDBSCAN hyperparameter sweep on UMAP-reduced DEB features.** **Panels (a)–(c)** Silhouette-score heatmaps over `min_cluster_size` (rows) and `min_samples` (columns) for three representative snapshots (t_{tr}, t_{mid}, t_{ss}). Each cell reports the silhouette of HDBSCAN on the fixed 2D UMAP embedding (`n_neighbors`=10, `min_dist`=0.0), restricted to parameter pairs that yield exactly two non-noise clusters (crystal vs. amorphous); cells that fail this criterion are left blank. **Panels (d)–(f)** UMAP scatter plots for the best scoring settings in Panels (a)–(c), with points colored by HDBSCAN label. The two phases are cleanly separated, with a thin boundary of points near the interface.

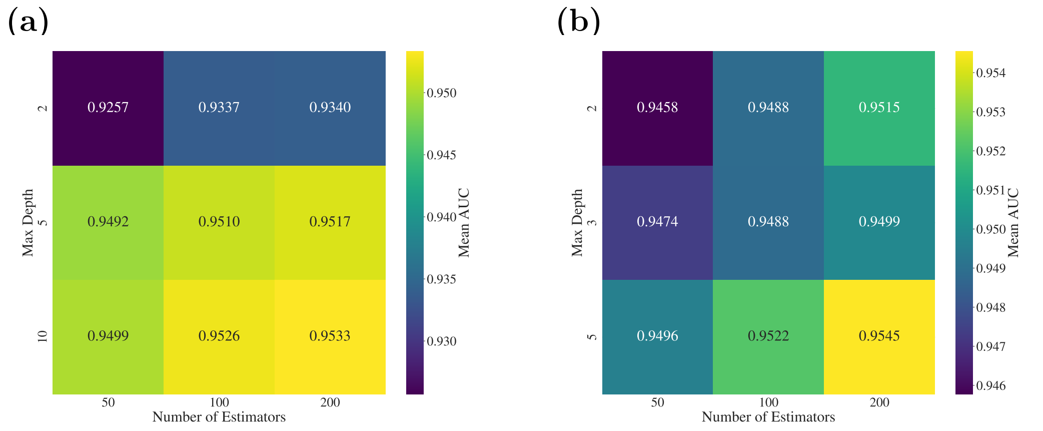

Figure S4: Hyperparameter grid search results for ensemble classifiers. **(a)** Random Forest and **(b)** Gradient Boosting classifiers. Each panel shows the mean test-set AUC across five stratified folds as a function of tree depth and the number of estimators. Both models exhibit smooth performance landscapes with consistently high accuracy (AUC $\gtrsim$ 0.95), demonstrating robustness to hyperparameter choice and supporting the use of shallow-to-moderate tree depths combined with sufficiently large ensemble sizes.

for detecting crystal–melt interfacial atoms. Consistent with this goal, all models achieve strong AUCs with balanced trade-offs among precision/recall (Fig. S5). The binary Logistic Regression models isolate different aspects of the interfacial decision: the *LR_ surface_ melt* task yields very high specificity to the melt phase with strong surface detection, while the *LR_ surface_ crystal* task is more challenging due to tighter feature proximity near crystal surfaces, which slightly reduces precision yet maintains competitive recall. The multiclass ensemble models (RF, GB) display high overall accuracy and well-calibrated decision boundaries, with most errors concentrated at the physically plausible interfaces (surface $\leftrightarrow$ melt or surface $\leftrightarrow$ crystal), as highlighted in the confusion matrices (Fig. S6).

Silver-standard ground truth. Surface labels are derived from *alpha-shape* boundaries computed on clusters identified by the C-index segmentation (see [2] for benchmarking details of the C-index). In brief, atoms belonging to the alpha-shape boundary (or within a small morphological band around it) are marked as *surface*, while interior atoms inherit *crystal* and

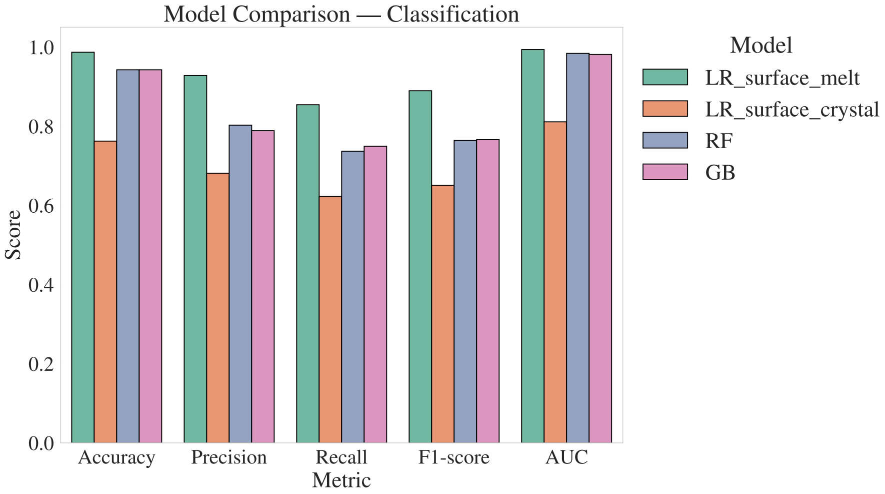

Figure S5: Model comparison across classification metrics. Mean cross-validated Accuracy, Precision, Recall, F1, and AUC are shown for Logistic Regression (*LR_ surface_ melt*, *LR_ surface_ crystal*), Random Forest (RF), and Gradient Boosting (GB). All models achieve high AUCs, indicating that DEB descriptors provide a strong signal for surface identification; differences reflect expected trade-offs between precision and recall at the crystal–melt interface.

exterior atoms *melt* labels. This silver-standard definition is geometrically grounded and aligns with the expected interfacial topology of crystal nuclei.

Overall, these results indicate that DEB descriptors encode interfacial structure robustly across model families. Differences between algorithms are modest relative to the consistently high AUCs, supporting the conclusion that *feature signal*—rather than classifier choice—is the primary driver of performance.

S3 Higher-MW demonstration (C500)

To illustrate transferability, we analyzed a representative snapshot from a higher-molecular-weight system (360 chains of C500) using the same DEB workflow. UMAP embeddings and phase overlays are shown in Fig. S7; DEB maintains clear crystal/melt separation and interfacial atoms in the transition state between the 2 state in the middle, consistent with the C150 system.

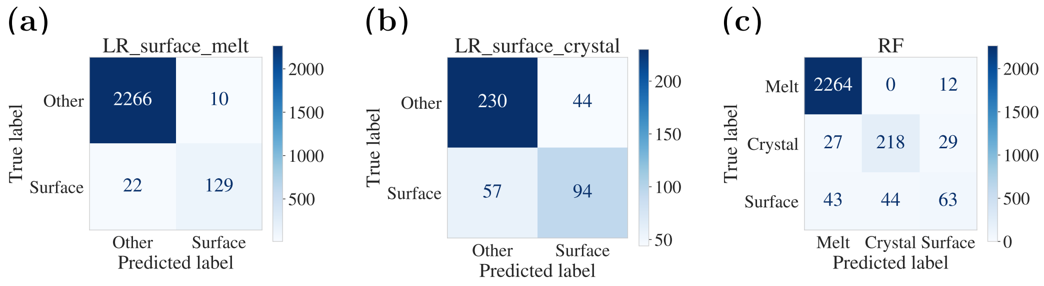

Figure S6: Representative confusion matrices. **(a)** Logistic Regression (surface vs. melt) and **(b)** Logistic Regression (surface vs. crystal) illustrate the class-specific behavior of the two binary tasks. **(c)** Random Forest (multi-class: melt/crystal/surface) demonstrates strong overall accuracy with most residual errors concentrated at interfacial classes (surface ↔ melt/crystal). These patterns are consistent with the physics of boundary atoms and corroborate the DEB feature signal.

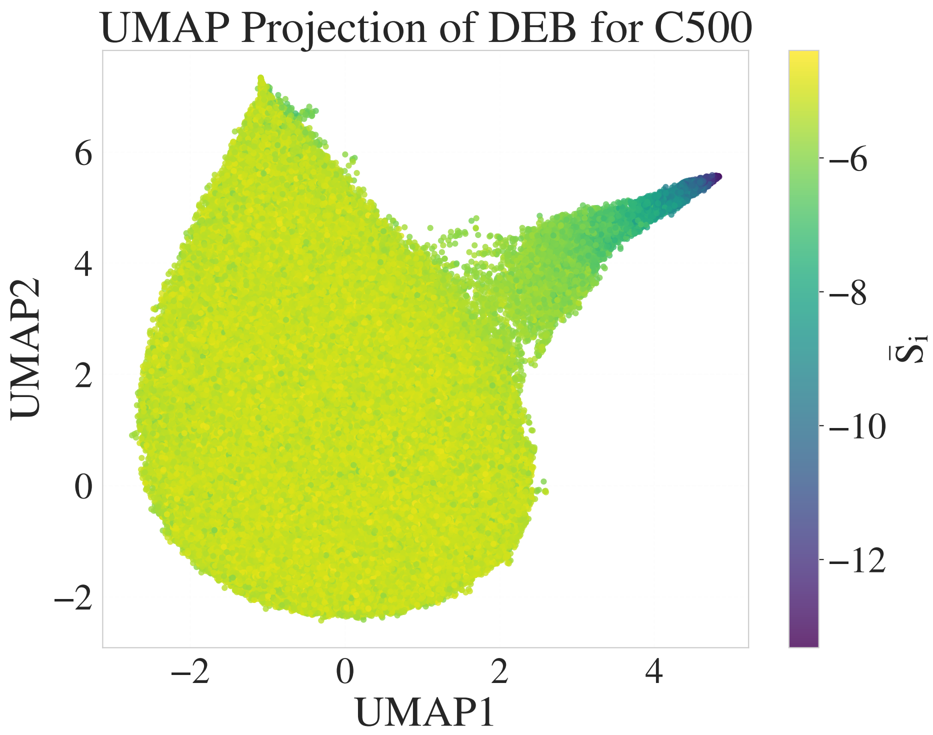

Figure S7: C500 demonstration: UMAP embedding with scaler entropy ($\bar{S}_i$) overlays using DEB features.

S4 Code and data bundle

A compressed archive `SM_DEB_SOAP.zip` accompanies this paper and contains: (i) descriptor datasets (DEB feature arrays, scalar entropy values, SOAP vectors) for the t_{mid} snapshot, (ii) scripts for UMAP/HDBSCAN, supervised classification (LogReg, RF, XGBoost), and plotting, A `README.txt` in the archive documents environment requirements and reproduction steps. The code is also available at our GitHub repository `https://github.com/etourani/DEB`. The GitHub version will be updated with any future extensions.

References

[1] Charu C. Aggarwal, Alexander Hinneburg, and Daniel A. Keim. On the surprising behavior of distance metrics in high dimensional space. In Jan Van den Bussche and Victor Vianu, editors, *Database Theory — ICDT 2001*, pages 420–434, Berlin, Heidelberg, 2001. Springer Berlin Heidelberg.

[2] Elyar Tourani, Brian J. Edwards, and Bamin Khomami. Machine learning workflow for analysis of high-dimensional order parameter space: A case study of polymer crystallization from molecular dynamics simulations, 2025.
